# Preventing the coffee-ring effect and aggregate sedimentation by *in situ* gelation of monodisperse materials†
†Electronic supplementary information (ESI) available. See DOI: 10.1039/c8sc03302a


**DOI:** 10.1039/c8sc03302a

**Published:** 2018-08-23

**Authors:** Huaiguang Li, Darren Buesen, Rhodri Williams, Joerg Henig, Stefanie Stapf, Kallol Mukherjee, Erik Freier, Wolfgang Lubitz, Martin Winkler, Thomas Happe, Nicolas Plumeré

**Affiliations:** a Center for Electrochemical Sciences (CES) , Faculty of Chemistry and Biochemistry , Ruhr University Bochum , Universitätsstr. 150 , D-44780 Bochum , Germany . Email: nicolas.plumere@rub.de; b Lehrstuhl für Physikalische Chemie II , Ruhr-Universität Bochum , 44780 Bochum , Germany; c Leibniz-Institut für Analytische Wissenschaften -ISAS- e.V. , 44227 Dortmund , Germany; d Max-Planck-Institut für Chemische Energiekonversion , Stiftstrasse 34-36 , 45470 Mülheim an der Ruhr , Germany; e Lehrstuhl Biochemie der Pflanzen , Ruhr-Universität Bochum , 44780 Bochum , Germany

## Abstract

Drop-casting and inkjet printing are virtually the most versatile and cost-effective methods for depositing active materials on surfaces. However, drawbacks associated with the coffee-ring effect, as well as uncontrolled aggregation of the coating materials, have impeded the use of these methods for applications requiring high control of film properties. We now report on a simple method based on covalent cross-linking of monodisperse materials that enables the formation of thin films with homogeneous thicknesses and macroscale cohesion. The coffee-ring effect is impeded by triggering gelation of the coating materials *via* a thioacetate–disulfide transition which counterbalances the capillary forces induced by evaporation. Aggregates are prevented by monodisperse building blocks that ensure that the resulting gel resists sedimentation until complete droplet drying. This combined strategy yields an unprecedented level of homogeneity in the resulting film thickness in the 100 nm to 10 μm range. Moreover, macroscale cohesion is preserved as evidenced by the long-range charge transfer within the matrix. We highlight the impact of this method with bioelectrocatalysts for H_2_ and NADPH oxidation. Peak catalytic performances are reached at about 10-fold lower catalyst loading compared to conventional approaches owing to the high control on film cohesion and thickness homogeneity, thus setting new benchmarks in catalyst utilization.

## Abstract 2

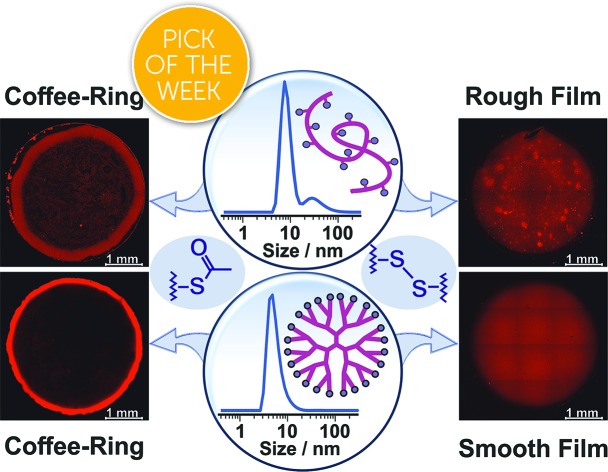
Microscale uniformity and long-range cohesion in multi-functional films assembled through drop-casting is realized by *in situ* gelation of monodisperse building blocks.

## Introduction

Drop-casting methods and ink-jet printing are attractive for the assembly of functional films owing to their scalability, their ease and speed of implementation, as well as their suitability for most types of surfaces and materials. However, the interplay of mass and heat transport processes taking place within the drying droplets leads to deposits which often considerably depart from the desired film morphology^1^ and thus restrain their applicability. In particular, the so-called “coffee-ring effect” (CRE) is omnipresent in drop-casting.^2^ It is due to solvent evaporation during film assembly that stimulates capillary flows within the drop that in turn displace particles to the three-phase contact line. As a result, particle accumulation at the dry film boundaries is accelerated, and a coffee-ring is formed.^3^ The width of the ring is often in the micrometer range, meaning that the major fraction of the area initially covered with the droplet remains mostly unmodified. Common solutions to circumvent the CRE^4^ rely on the engineering of the properties of the droplet,^5^ of the surface,^6^ of the solvent and co-solvent,^7^,^8^ or of the particles to be deposited.^9^–^11^ In particular, aggregate sedimentation induced by physical interparticle agglomeration, addition of gelling agents,^12^,^13^ chemical or photochemical cross-linking^7^,^14^ can be used to promote vertical deposition while suppressing radial flow and thus a more uniform deposit is formed.

However, the properties of drop-cast films containing additives or made of aggregates often suffer from insufficient cohesion, homogeneity and film thickness uniformity. These requirements are fundamental for numerous applications based on active films such as organic semiconductors^15^ for photovoltaics,^16^ inorganic catalysts for water splitting,^17^ or (bio-)molecular catalysts for sensing^18^ and energy conversion.^19^,^20^ The thickness of the film is of particular importance since it defines the factors governing the catalytic current or photocurrent generation.^21^–^24^ Therefore, if the thickness is heterogeneous because of the presence of aggregates, some parts of the film will have sub-optimal dimensions which will be detrimental to the overall catalytic or photoactive performances. Moreover, breaks in matrix structure due to aggregate boundaries would interrupt key features such as long-range charge transfer pathways in electrocatalytic/photovoltaic films,^25^ energy transfer in light emitting systems,^26^ plasmonic effects in macroscopic 3D superlattices^11^ or network integrity in macroscale stimuli responsive materials.^27^ For these reasons, methods for reproducible formation of films with controlled and nearly homogeneous thicknesses as well as macroscale interconnection are highly desirable to enable their practical applications.

**Fig. 1:**
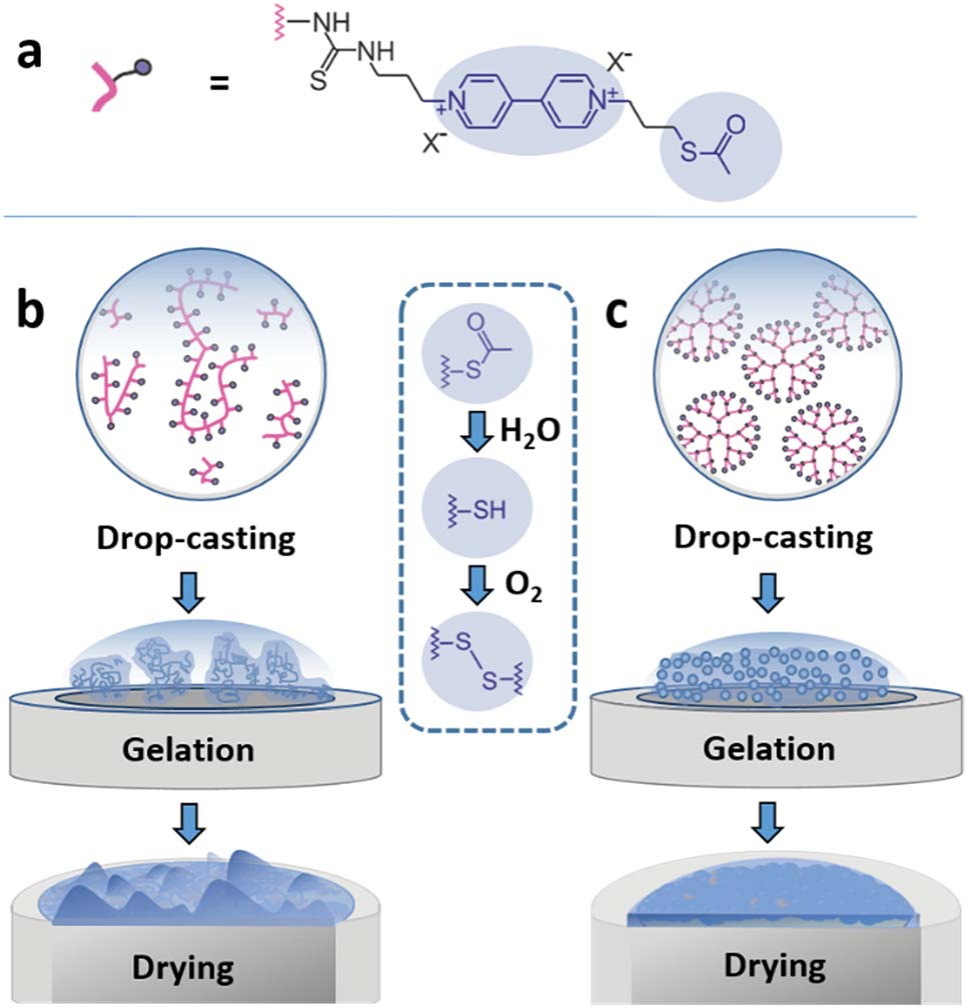
Film assembly by drop-casting and *in situ* gelation. (a) The macromolecules are modified with a viologen moiety, serving as a dual redox/fluorescent functionality, and terminated with a thioacetate group for crosslinking. (b) Drop-casting of the polydisperse viologen-modified polymer results in a rough film due to aggregate precipitation during gelling and drying. (c) Drop-casting of the monodisperse viologen-modified dendrimer yields a homogeneous gel which deposits evenly upon droplet drying. The blue inset in the center shows the hydrolysis of the thioacetate group to –SH groups followed by oxidation of the latter to disulfide bonds resulting in crosslinking and gelation.

Here, we demonstrate that drop-casting of monodisperse building blocks followed by *in situ* gelation prior to complete droplet drying circumvents both coffee-ring effects and aggregate formation. We take advantage of a mild crosslinking chemistry based on thioacetate terminated macromolecules^19^,^21^,^28^ to induce gelation. The coating materials based either on polymeric or on dendrimeric scaffolds were functionalized with viologen moieties serving both as redox relays and fluorescence reporters (Fig. 1a). The key factor enabling the formation of uniform and highly cohesive films is the ability to maintain gel homogeneity during droplet drying. While drop-casting and gelation of polydisperse polymeric materials efficiently suppresses the CRE, the sedimentation of polymer aggregates that are larger than the nominal thickness of the resulting film leads to excessive thickness distributions (Fig. 1b). In contrast, the use of dendrimers as monodisperse building blocks in place of polydisperse macromolecules results in films that have highly homogeneous thicknesses and that are free of breaks in their macroscale 3D matrix as evidenced by the electron accessibility throughout the complete volume of the film (Fig. 1c). The applicability of the dendrimer hydrogel films as the immobilization matrix is verified for bioelectrocatalytic systems, namely hydrogenase and ferredoxin-NADP^+^-reductase (FNR), which both yielded high current densities and excellent stability under constant turnover for H_2_ oxidation and NADPH oxidation, respectively.

## Results and discussion

### Structure of the viologen-modified macromolecules

The viologen-modified dendrimer (Scheme S1, ESI†) was synthesized starting from a polyamidoamine (PAMAM) core (3^rd^ generation, 32 head groups). The synthesis of the viologen-modified polymer was based on a branched polyethylenimine (PEI) backbone according to a previously reported procedure.^19^ In both cases, the same thioacetate terminated viologen groups were used for post-functionalization (Fig. 1a). The high density of primary amine groups on the PAMAM dendrimer and on the PEI backbone enables high loadings with viologen moieties upon covalent attachment *via* isothiocyanate chemistry (Scheme S2, ESI†). ^1^H NMR characterization of the viologen-modified dendrimer demonstrates almost quantitative functionalization (99% yield, Fig. S1, ESI†). A similarly high post-functionalization yield is achieved for the viologen-modified polymer according to UV-Vis measurements (Fig. S2, ESI†).

### Spontaneous crosslinking and gelation

The thioacetate termination on the viologen head groups serves as a crosslinking functionality for *in situ* gelation under mild conditions (Fig. 1). The thioacetate hydrolyzes in aqueous solutions,^29^ and the resulting free thiol groups subsequently oxidize in the presence of O_2_ to form disulfide bonds, leading to crosslinking between the macromolecules. Moderately basic buffers (pH 9) were used to accelerate both the thioacetate hydrolysis and the thiol oxidation reactions. Gelation of the viologen-modified macromolecules was investigated in solution by dynamic light scattering (DLS), which is well suited for monitoring changes in macromolecule size and size distributions without disturbing the gelling system.^30^

**Fig. 2:**
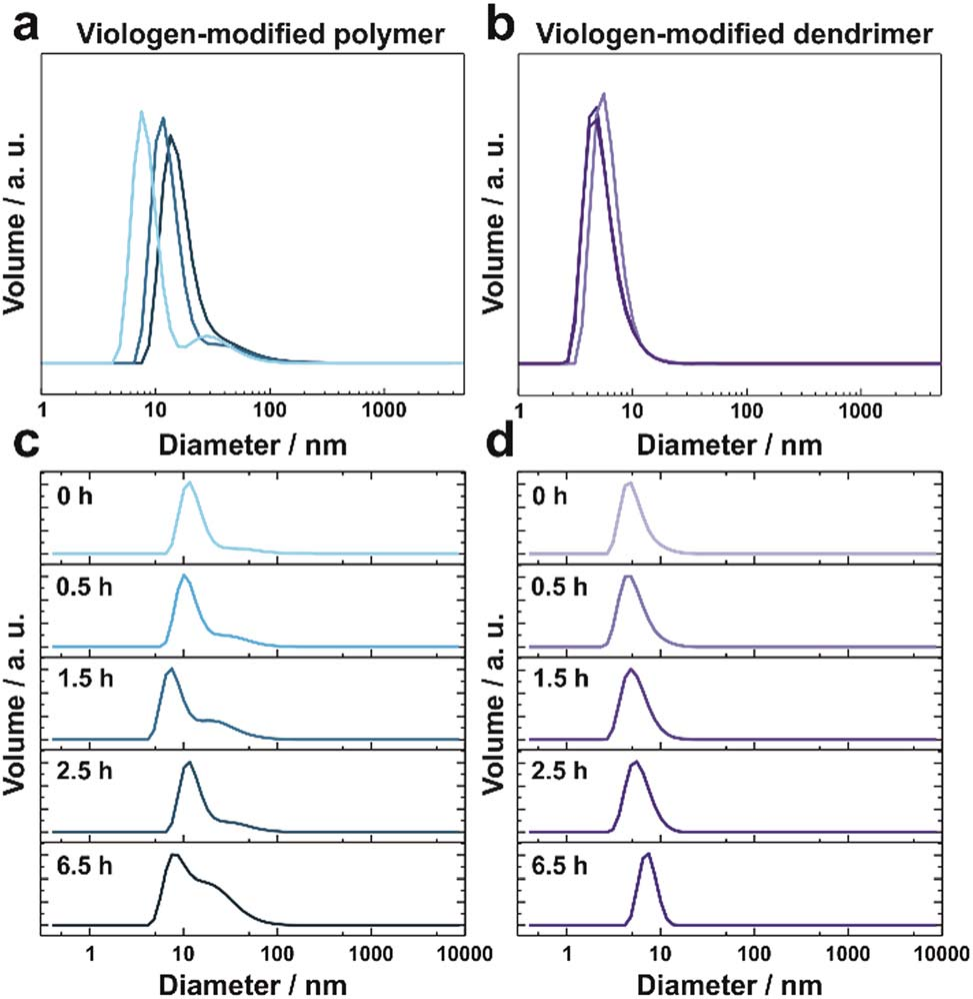
Size distributions of viologen-modified dendrimers and polymers. Dynamic light scattering measurements of (a) viologen-modified polymer and (b) viologen-modified dendrimer under anaerobic conditions. The three replicates were from three individual preparations under the same conditions. (c) Viologen-modified polymer and (d) viologen-modified dendrimer after various incubation times (as indicated in the figure) in ambient air. The concentrations of the polymer and dendrimer are 0.25 mg mL^–1^ and 1 mg mL^–1^, respectively. DLS data are presented as distribution by volume. All measurements were performed in Tris buffer solution (100 mM, pH 9.0), in the presence of tris(2-carboxyethyl)phosphine (TCEP, 1.25 mM).

Under anaerobic and reducing conditions, the viologen-modified polymer shows a high polydispersity with the main fraction of coil size in the 10–30 nm range, as well as presence of aggregates in the 100–500 nm (0.6%) and 3–4 μm (0.2%) range (Fig. 2a). In contrast, the dendrimers display an average diameter of 5.57 ± 0.04 nm (Fig. 2b) with very low distribution which is in agreement with the nanoscale dimension^31^ and molecular nature of the viologen-modified dendrimer.

When the solutions are exposed to air, the average size for both the polymer and the dendrimer increases due to crosslinking *via* oxidation of the thiol groups by O_2_ (Fig. 2c and d). After 6.5 h, the polymer displays a broadening of the main peak as well as an increase of the shoulder in the range from 30 nm to 50 nm (Fig. 2c). In the case of the dendrimer, only a very small fraction of particles (<0.2%) with a diameter of a few hundred nanometers appears after 6.5 h, and the main peak shows an increase in particle diameter from around 5 nm to 8 nm, while maintaining a very low polydispersity (Fig. 2d). This indicates that the monodisperse dendrimeric systems undergo crosslinking without significant aggregation in contrast to the gelation of the branched polymeric materials. The absence of large particles in the DLS of the crosslinked dendrimers implies the formation of a homogeneous gel. The relatively long time needed for gelation for both macromolecules under the DLS conditions is attributed to slow diffusion of O_2_ into the large volume of the DLS cuvette (2 mL) and to the time needed for initial neutralization of the reducing agent (tris(2-carboxyethyl)phosphine, TCEP).

### Hydrogel film formation and film morphology

**Fig. 3:**
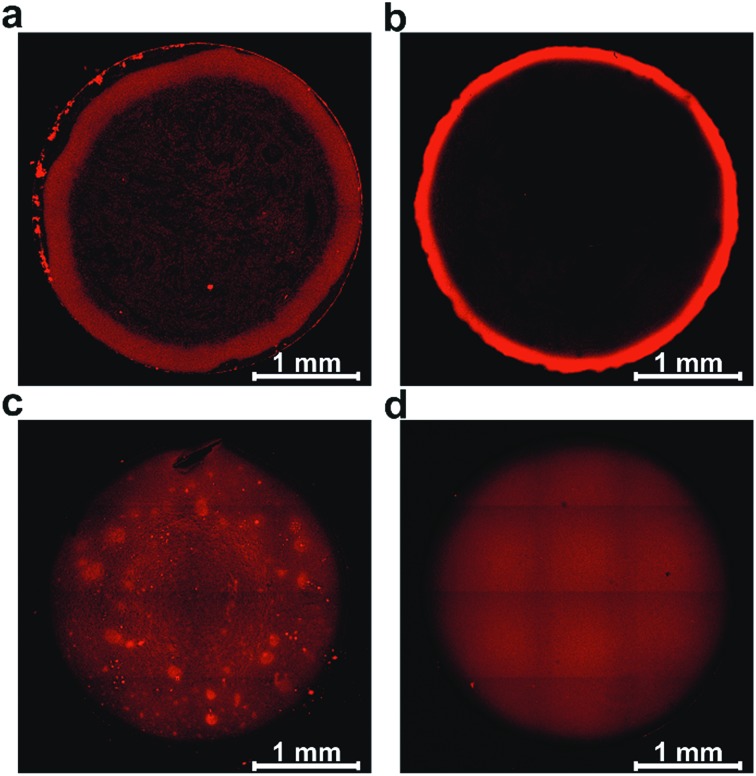
Effect of crosslinking and polydispersity on coffee ring and aggregate formation. Fluorescence microscopy images of films assembled by drop-casting of the viologen-modified macromolecules: (a) polymer without crosslinking, (b) dendrimer without crosslinking, (c) polymer with crosslinking and (d) dendrimer with crosslinking. Fluorescence is induced by excitation at 488 nm and emission signals are collected from 500 nm to 650 nm. In all cases, the glassy carbon disks (3 mm in diameter) were modified with a surface coverage of 200 μg cm^–2^. For (a) and (b) excess TCEP was used to prevent crosslinking. For (c) and (d) the gelation process was carried out prior to droplet drying with a total volume of 2.5 μL, including 0.5 μL Tris buffer solution (100 mM, pH 9.0) at RT in a water saturated atmosphere.

Film formation was performed by drop-casting microliter sized droplets (1 to 4 μL) onto glassy carbon surfaces. Fluorescence microscopy was used to qualitatively study the effect of drop-casting conditions on film morphology (Fig. 3). Fluorescence arises from the excitation of the viologen moieties. The measurements were performed in a confocal setup so that fluorescence intensities are related to the local concentrations and local thicknesses of the viologen modified films (see Methods section). In a first set of experiments, the crosslinking of the macromolecules was hindered by using an excess concentration of TCEP as the reducing agent to prevent disulfide bond formation. The fluorescence microscopy images of the films derived from the polymer and from the dendrimer both display ring-shaped structures with intensive fluorescence emission on the periphery of the films (Fig. 3a and b). These are the typical coffee-ring features which result from the transport of the macromolecules to the three phase boundary of the droplet due to the capillary forces induced by solvent evaporation.^7^

In a subsequent set of experiments, the concentration of the reducing agent was lowered so that crosslinking can take place before droplet drying. The low droplet volume combined with the large air–solution interfacial area allows for fast saturation of the solution with O_2_, ensuring a rapid establishment of oxidative conditions which induces the crosslinking *via* disulfide bond formation. The fluorescence microscopy images reveal that the resulting films now cover the complete drop-cast area and the coffee rings are fully suppressed (Fig. 3c and d). This demonstrates that the crosslinking *via* spontaneous disulfide bond formation results in a material that is unaffected by the capillary forces induced by droplet drying. Remarkably, the disulfide crosslinking eliminates the coffee-ring effect even in the case of the dendrimer molecules despite their spherical shape which is particularly prone to undergo coffee-ring deposition.^9^ The second important observation is that the morphologies of the films based on the monodispersed dendrimer are highly uniform in contrast to the polymer based films which exhibit numerous and large aggregates. This is in agreement with the respective gelation behavior observed for the two materials in the DLS investigation. The aggregates of polymers precipitate and thus yield a rough film while the dendrimer gel remains in solution and deposits evenly upon complete droplet-drying. Remarkably, the smoothness of the latter film matches previously reported systems obtained by the addition of a gelling agent.^13^ The ability to achieve film homogeneity without additives is a valuable feature of the present approach since it ensures that the film composition can be entirely defined by the intended application rather than the parameters required to obtain uniform deposits.

**Fig. 4:**
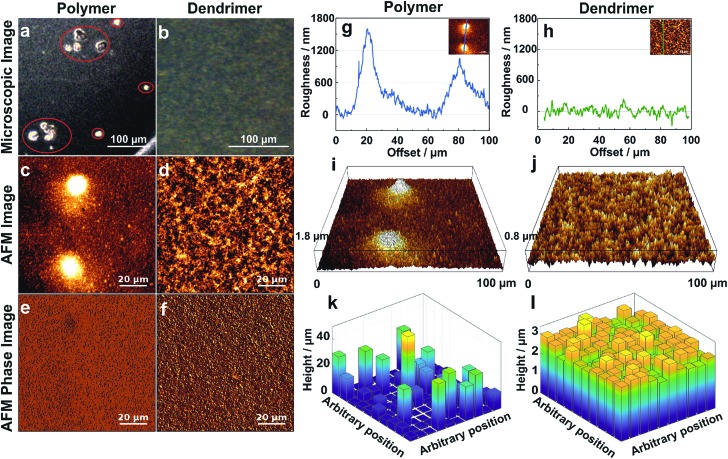
Morphology of the hydrogel films assembled by drop-casting: viologen-modified polymers (a, c, e, g, i and k) and viologen-modified dendrimers (b, d, f, h, j and l) on the surface of glassy carbon disks (3 mm in diameter). (a and b) Optical microscopy images (aggregates are highlighted in red). (c and d) AFM images showing the surface morphology over 100 μm × 100 μm areas. (e and f) Phase analysis of AFM images of (c) and (d). (g and h) Surface roughness of the film derived from the crossline over the AFM images. The inset images illustrate the position of the respective crossline. (i and j) 3D AFM images derived from c and d, respectively. (k and l) 3D representations of the films extracted by the electrochemical method for determination of the thickness distributions. The film sections have arbitrary positions. The corresponding electrochemical data, secondary plots and probability distribution functions are given in Fig. S4 (ESI†). For both the polymer and the dendrimer, the glassy carbon disks were modified with a surface coverage of 200 μg cm^–2^ and the gelation process was carried out with a total volume of 2.5 μL, including 0.5 μL Tris buffer solution (100 mM, pH 9.0) at RT in a water saturated atmosphere.

The effect of aggregation on the morphology of the film is further characterized by optical and atomic force microscopy (AFM). The optical microscopy images of the films resulting from drop-casting of viologen-modified polymers display numerous aggregates having diameters in the 20–50 μm range (Fig. 4a), whereas the images of the films of viologen-modified dendrimers are relatively smooth without any visible aggregates over an equally large sampling area (Fig. 4b). These observations corroborate the fluorescence microscopy investigations. AFM imaging of the polymer films (Fig. 4c) coupled to phase analysis (Fig. 4e) reveals that the aggregates and the relatively smooth adjacent regions are of the same composition, implying full but uneven coverage of the surface by the polymer film. These aggregates are in the micrometer magnitude according to the surface roughness measurements (Fig. 4g and i). In contrast, the AFM images of the dendrimer film display a uniform, smooth surface over an area of 100 μm × 100 μm (Fig. 4d). Phase analysis, surface roughness and 3D images also confirm the homogeneous coverage with the dendrimer film over the complete sampled area (Fig. 4f, h and j). The mean thickness of the dendrimer film obtained by AFM upon scratching of the surface was found to be (3.5 ± 0.3) μm and was consistent for variable locations on the electrode surface (Fig. S3, ESI†).

### Electroactive film thickness distribution

The electroactive thickness distributions of the films were subsequently investigated through cyclic voltammetry.^32^ The main benefit of using an electroanalytical method in comparison to AFM is that it probes the portion of the film that is accessible to electrons and therefore, the portion of the film matrix that is break-free and able to contribute to electrochemical processes. Additionally, the whole surface of the electrode is probed instead of a selected area. In this electrochemical method, cyclic voltammograms (CVs) of the redox film are measured at different experimental time scales defined by the scan rates (*ν*) (Fig. S4A and B, ESI†), and the normalized peak currents (*i*_p_/*ν*^1/2^) are plotted *versus ν*^1/2^. The experimental *i*_p_/*ν*^1/2^*vs. ν*^1/2^ curve for the dendrimer film shows only minor deviations from the theoretical current response for a perfectly smooth film which indicates a highly uniform thickness distribution (Fig. S4D, ESI†). In comparison, the corresponding dimensionless peak current plots for the viologen-modified polymer substantially deviate from the curve corresponding to a perfectly homogeneous film, implying that the polymer films are highly aggregated (Fig. S4C, ESI†). Probability distribution functions of the film thickness for the polymer and the dendrimer films (Fig. S4E and F, ESI†) were extracted by analysis of the deviations from the theoretical *i*_p_/*ν*^1/2^*vs. ν*^1/2^ curves, and 3D representations of these films were generated with arbitrary positions of the individual film sub-sections (Fig. 4k and l). The relative standard deviations (*σ*), expressed as a percentage of the mean, were determined for the dendrimer and polymer films based on the underlying film thickness distribution, and were found to be 10.7% and 169% respectively. The 3D representation of the polymer film (Fig. 4k) illustrates the presence of the micrometer scaled aggregates, which are in good general agreement with the one observed in the optical and AFM images (Fig. 4a and g). In contrast, the 3D representation of the dendrimer film shows a very smooth surface in which both “coffee rings” and aggregates are absent (Fig. 4l). The high degree of uniformity of the film thickness distribution and the average thickness (2.6 μm) obtained from the electrochemical method are in quantitative agreement with the corresponding AFM investigations, which indicates that the entire redox-active film is accessible to electrochemical processes, and thus, that the electron transfer pathway within the complete film volume is free of blockages or breaks.

### Effect of gelation conditions on film homogeneity

**Fig. 5:**
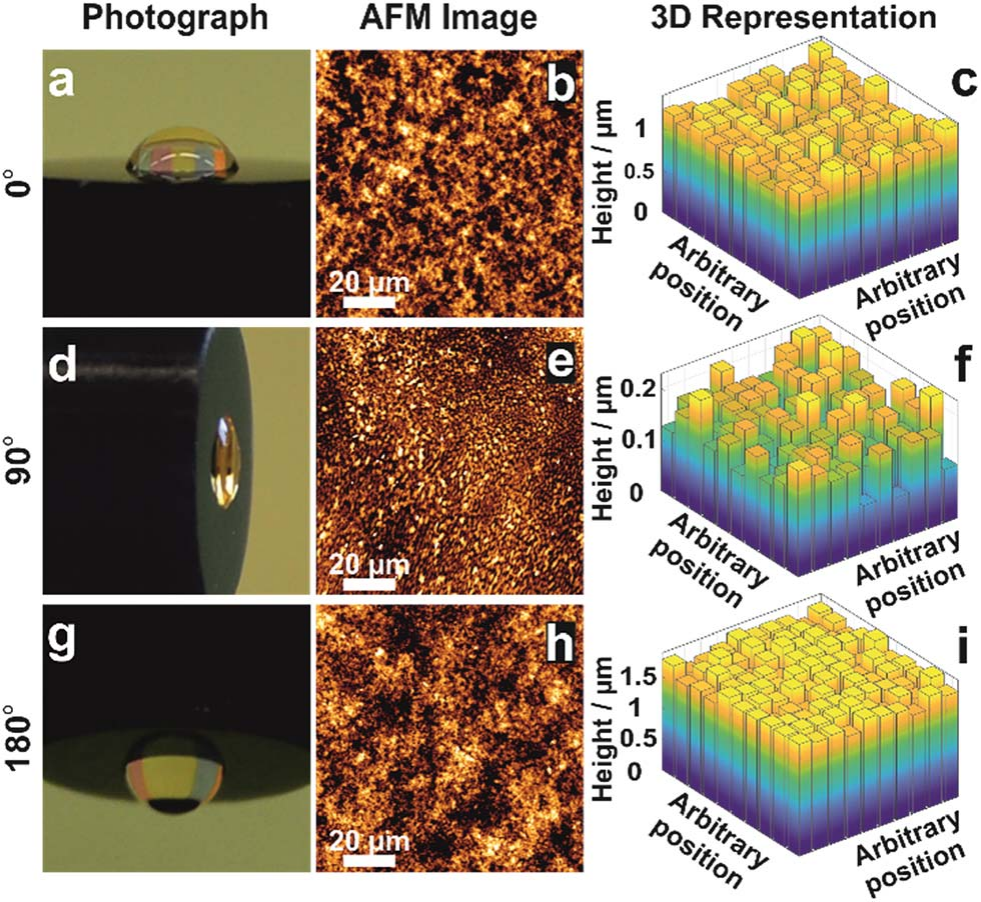
Effect of tilt angle during droplet drying on film thickness distribution. Electrodes modified with viologen-modified dendrimers from a droplet dried on a gold disk (2 mm in diameter) with tilt angles of (a) 0°, (d) 90° and (g) 180°. Film morphology from AFM images for tilt angles of (b) 0°, (e) 90° and (h) 180°. 3D representations derived from the electrochemical method for determination of the film thickness distribution for tilt angles of (c) 0°, (f) 90° and (i) 180°. The corresponding electrochemical data, secondary plots and probability distribution functions are given in Fig. S6–S8 (ESI†). The droplet volume for film formation was 2 μL for the tilt angles of 0° and 180°, and was 0.5 μL for the 90° tilt angle. The smaller droplet size in the latter case was chosen to prevent droplet deformation due to gravity.

The morphology of films obtained from the precipitation of a colloidal solution within a drying droplet generally depends on the wetting properties of the surface^33^ as well as on its tilt angle.^34^ In particular, the coffee ring effects are exacerbated for pendant droplets and on hydrophobic surfaces. To test the general applicability of the *in situ* gelation method for uniform film formation presented in this work, we compared the assembly of the dendrimer based hydrogel films on gold and carbon surfaces. Optical microscopy (Fig. S5A and B, ESI†) and AFM images (Fig. S5C and D, ESI†) show films of low roughness on both Au and C substrates. The film thickness distributions extracted from electrochemical investigations (Fig. S5E–H, ESI†) are low on both materials (*σ*_Au_ = 8.7% and *σ*_C_ = 10.7%). This demonstrates that interactions between the materials for deposition and the surface of the substrate do not significantly impact the resulting film thickness homogeneity. Additionally, film preparations were peformed film preparations on Au surfaces with different tilt angles (Fig. 5) to validate the mechanism for uniform film formation. The film resulting from a pendant droplet (180°), as well as the film from a droplet sticking to a vertically orientated surface (90°), is highly homogeneous and comparable to the one obtained from a sessile droplet (0°). The low thickness distributions obtained for these films (*σ*_0°_ = 8.7%, *σ*_90°_ = 16.8% and *σ*_180°_ = 4.8%) regardless of the orientation and surface properties indicate that the film formation is not a result of aggregation and sedimentation of the cross-linked macromolecules. This corroborates that the mechanism is based on *in situ* gelation of the dendrimers followed by even deposition of the resulting hydrogel onto the surface as it dries.

### Electrocatalytic applications

**Fig. 6:**
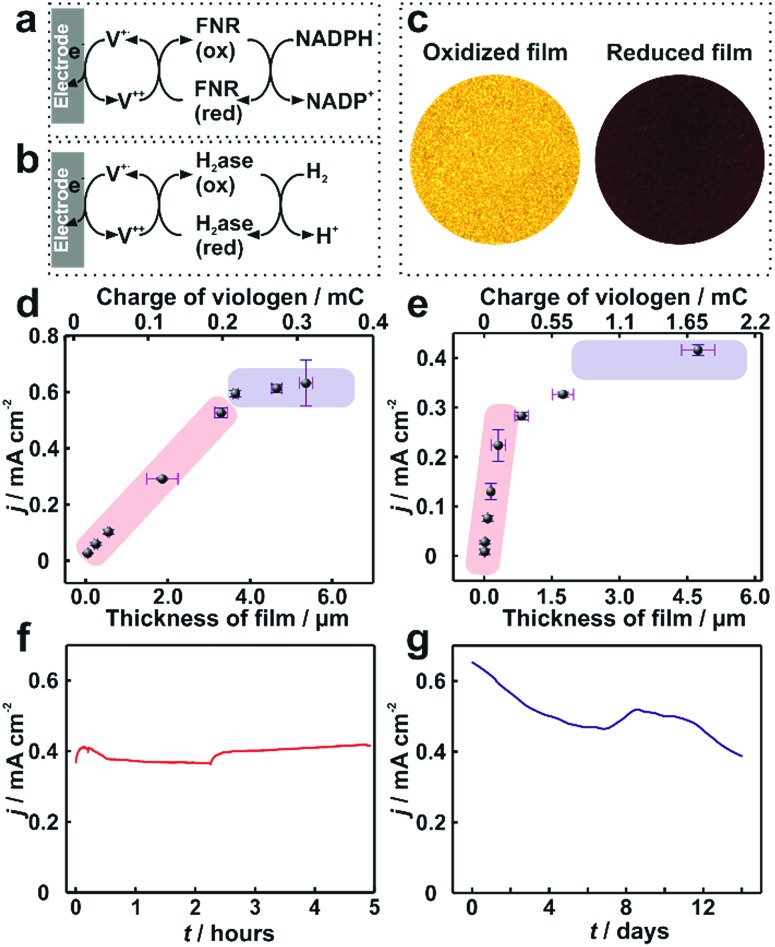
Catalytic current density for ferredoxin-NADP^+^-reductase (FNR) and hydrogenase immobilized in viologen-modified dendrimer films. Electron transfer schemes for (a) FNR and (b) hydrogenase immobilized in viologen modified films. (c) Optical microscopy images of the hydrogenase–dendrimer film on Au electrodes in oxidized (left) and reduced states (right). The reduced state was obtained in aqueous Na_2_S_2_O_4_ (0.1 M). Catalytic current density (*j*) *vs.* film thickness (*l*) for (d) NADPH oxidation with immobilized FNR (the corresponding CVs are given in Fig. S10, ESI†) and (e) for H_2_ oxidation with immobilized hydrogenase. Long-term catalytic current measurements of the (f) dendrimer–FNR electrode (*l* = 2 μm) in NADPH solutions and (g) dendrimer–hydrogenase electrode (*l* = 4.1 μm) in 100% H_2_. The fluctuations in the catalytic currents correspond to changes in stirring rate (in (f)) and to buffer replacement (in (g)). All measurements with FNR modified electrodes were performed in phosphate buffer (pH 7.2, 0.1 M) containing NADPH (40 mM). For (f), vigorous stirring and a constant applied potential of –0.3 V *vs.* Ag/AgCl (3 M KCl) were used. All dendrimer–hydrogenase electrodes were measured under 100% H_2_ in phosphate buffer (pH 7.0, 0.1 M) at 2000 rpm. In (g), the potential was held at 0 V *vs.* Ag/AgCl (3 M KCl). The standard deviations were obtained from two replicates from two individual preparations under the same conditions. All measurements were conducted under anaerobic conditions.

In electrocatalytic applications, film thickness homogeneity and electron transfer throughout the entire volume of the film are important factors that define the resulting catalytic performance. The applicability of the dendrimer based hydrogel was tested for NADPH oxidation catalyzed by the redox enzyme ferredoxin-NADP^+^-oxidoreductase (FNR) (Fig. 6a) as well as for H_2_ oxidation catalyzed by the metallo-enzyme *Desulfovibrio vulgaris* MF (*Dv*MF) NiFe-hydrogenase (Fig. 6b). In both cases, the viologen moities serve as electron relays between the electrode and the redox enzymes within the hydrogel film. The bioelectrocatalytic films were prepared by drop-casting a mixture of dendrimer and of the respective enzyme on Au electrodes. The viologen moieties confer the redox properties to the film, which appears colorless in the oxidized state and dark blue in the reduced state (Fig. 6c). Long-range charge transfer takes place through electron hopping between viologens and is measured as an apparent electron diffusion coefficient (*D*_e_).^25^ The value of *D*_e_ for the dendrimer films is (1.15 ± 0.1) × 10^–8^ cm^2^ s^–1^ (see Fig. S9, ESI†), which is about twice as high as the *D*_e_ value obtained for viologen-modified polymer films ((4.7 ± 1.7) × 10^–9^ cm^2^ s^–1^).^21^

For FNR immobilized in dendrimer modified films, the catalytic current for NADPH oxidation reaches a maximum of 0.65 mA cm^–2^ (Fig. 6d). Similarly, the hydrogenase immobilized in the dendrimer films delivers catalytic current density up to 0.4 mA cm^–2^ for H_2_ oxidation (Fig. 6e). In contrast, the use of viologen-modified polymer instead of dendrimer as the building block for the formation of films containing FNR resulted in maximum catalytic currents for NADPH oxidation of only 0.1 ± 0.04 mA cm^–2^ (Fig. S11, ESI†). This 6-fold lower current is rationalized not only by the lower *D*_e_ value in the polymer, but also by the aggregates present in the polymer film, which results in breaks in the electron transfer pathway and electrochemical inaccessibility of the catalysts.

Moreover, the film thickness (*l*) defines the factors limiting the catalytic current (*i*_cat_). Using theoretical models which account for the reaction–diffusion processes at play for a given set of film parameters,^21^–^24^ predictions of *i*_cat_*vs. l* can be constructed. For thin films, catalyst loading limits the current, and therefore, *i*_cat_*vs. l* is linear with a slope defined by the catalytic properties (enzyme concentration *C*_E_ and catalytic rate constants *k*_A_) according to eqn (1).^22^ For thicker films, electron hopping within the film typically becomes limiting and therefore a plateau in *i*_cat_ is expected, reaching a value which depends on the electron transfer properties according to eqn (2).^22^

1 *i*_cat_ = *nFAC*_A_*k*_A_*C*_E_ × *l*

2 *i*_cat_ = *nFAD*_e_^1/2^*C*_A_*k*_A_^1/2^*C*_E_^1/2^

where *A*, *n*, *F* and *C*_A_ are the electrode surface area, the number of electrons transferred, the Faraday constant, and the concentration of the electron mediator (the viologen moiety) within the film, respectively. Additional limiting cases related to mass transport or enzyme kinetics may also arise.^22^

If the thickness is heterogeneous, some parts of the film will be in one limiting regime, whereas other parts of the film will be in another limiting regime which will result in *i*_cat_*vs. l* behaviors that deviate from the theoretical predictions. Hence the *i*_cat_*vs. l* plots can be used to qualitatively assess the heterogeneity of the fraction of the film that is electrocatalytically active. Previous reports have shown that the use of polymers enables the formation of films with redox enzymes which display behaviors following eqn (1) or (2) but typically require more complex procedures, such as layer-by-layer assembly.^22^,^35^ In contrast, such control on bioelectrocatalytic response through film thickness adjustment has never been reported through simple drop-casting of redox-active polymers.

In the case of drop-casting of dendrimers on electrode surfaces, the average film thicknesses can be adjusted with the amount of the dendrimer and enzyme used for film formation (Fig. S12, ESI†). Remarkably, trends for *i*_cat_*vs. l* of the modified electrodes are in agreement with the behavior expected from the theoretical reaction/diffusion models for electrocatalytic films (eqn (1) and (2)).^22^,^23^ For FNR immobilized in a dendrimer matrix, the catalytic current for NADPH oxidation linearly scales with the film thickness (*l*) up to approximately 3.5 μm and subsequently transitions to a plateau (Fig. 6d). A similar behavior is observed for catalytic current for H_2_ oxidation obtained from hydrogenase modified electrodes (Fig. 6e). The clear experimental transition from a catalyst loading limitation in the thin film regime (linear region) to a limitation by electron transfer as the film thickness increases (plateau region) confirms that the use of viologen-modified dendrimers enables the formation of bio-electrocatalytic films with controlled and uniform thicknesses, and therefore allowing for the optimal utilization of the redox catalyst.

The long term stability of dendrimer modified films for bioelectrochemical processes within viologen-modified dendrimer/enzyme electrodes was investigated under conditions of continuous turn-over. These conditions were set by applying a constant potential, which induced the oxidation of the substrates (NADPH or H_2_) over long time periods ranging from hours to weeks (Fig. 6f and g). The catalytic current for the dendrimer–FNR electrodes remained completely stable within the first 5 hours of continuous NADPH oxidation. The dendrimer–hydrogenase electrode was maintained under continuous turnover conditions for H_2_ oxidation for 2 weeks, which resulted in only a 39% decrease in current. In addition, considering that the catalytic current is recorded under strongly oxidizing conditions by applying a potential of 0 V *vs.* Ag/AgCl at the electrode, which are deactivating for NiFe-hydrogenases,^36^ the stability of the current in the dendrimer modified films exemplifies how the hydrogenase–dendrimer films remain immune to this oxidative stress owing to the Nernst buffering properties of the viologen containing matrix.^19^,^28^ The stability and bioelectrocatalytic performances obtained for the thin dendrimer films (2 μm for the FNR-modified electrode and 4.1 μm for the hydrogenase-modified electrode) compete with the one previously reported for the viologen-modified polymer films with comparably much higher thicknesses (100–300 μm).^21^

## Conclusions

The crosslinking and gelation of monodisperse materials open up the possibility to use drop-casting methods for the assembly of functional films with a high degree of homogeneity and fine control of the film thickness by circumventing aggregate sedimentation and coffee-ring effects. Dynamic light scattering, fluorescence, optical and AFM imaging, as well as electrochemical and electrocatalytic investigations, support a mechanism for film formation involving in-droplet gelation followed by homogeneous deposition on the complete drop-cast area. Aggregate formation, which is intrinsic to classical methods based on precipitation processes used for coffee-ring suppression, is completely absent when using *in situ* gelation of dendrimeric materials as building blocks. Importantly, the cohesion of the film, as demonstrated by the long-range charge transfer pathways accessing the full volume of the film matrix, enables the optimal exploitation of the immobilized catalysts with performance in agreement with theoretical prediction for perfectly homogeneous films. Although the present study was based on dendrimeric materials, the underlying film formation mechanism allows extrapolation of the applicability of this drop-casting concept to any materials that enable homogeneous gel formation before droplet drying.

Moreover, from a practical perspective, a remarkable feature of this drop-casting method is that the resulting film thickness homogeneity is independent of the nature or tilt angle of the surface and the mild crosslinking conditions are compatible even for highly sensitive catalytic systems such as redox enzymes. As such, we anticipate that the concept will serve as a general basis for film formation of various active materials for applications ranging from electrocatalysis to organic photovoltaics or organic electronics. To this end, the extension from drop-casting to inkjet printing^1^ of monodispersed building blocks will be particularly valuable for small surface area coating or non-flat surfaces that are not accessible to classical deposition methods as well as for low-cost, industrial-scale printing for applications requiring a high degree of film homogeneity.

## Experimental section

### Materials and methods

Unless stated otherwise, all reagents used in the experiments were purchased from Sigma-Aldrich. β-Nicotinamide adenine dinucleotide phosphate tetrasodium salt (NADPH) was purchased from GERBU Biotechnik GmbH. All the materials were directly used as received without further purification.

### Synthesis of PAMAM G3 dendrimer with thioacetate functionalized viologen terminal groups (Schemes S1 and S2, ESI†)

PAMAM G3 dendrimer in methanol solution (290 μL, 7.2 μmol) was placed in an oven-dried Schlenk tube fitted with a stir bar, and the methanol was removed under vacuum. The resulting viscous oil was dissolved in dry DMSO (5 mL) and degassed with several cycles of vacuum and argon. After addition of isocyanate terminated viologen^19^ (1.5 equivalents, 201 mg, 347.4 μmol), the solution turned deep blue. The mixture was then stirred at room temperature for three days. After the mixture was decanted into an aqueous solution of KNO_3_ (0.1 M, 150 mL), the solution instantly turned yellow-green. The KNO_3_ solution was placed into a filtration unit fitted with a 5 kDa size exclusion membrane, and was filtered overnight with distilled water. The water was then removed under reduced pressure resulting in a glassy, dark green solid (145 mg, 88%).

^1^H NMR (400 MHz, D_2_O) *δ* 9.32–9.17 (m), 8.67 (d, *J* = 5.9 Hz), 4.95–4.85 (m), 3.90–3.48 (m), 3.44 (s), 3.48–3.29 (m), 3.07 (t, *J* = 6.7 Hz), 2.94 (s, br), 2.82 (s, DMSO), 2.75 (s, br), 2.58–2.42 (m), 2.47 (s) ppm. ^13^C NMR (101 MHz, D_2_O) *δ* 195.04, 169.34, 168.85, 144.71, 140.23, 121.87, 121.63, 55.16, 54.25, 45.97, 43.80, 43.50, 33.40, 31.31, 27.15, 24.94, 24.63, 19.61 ppm.

### Quantification of the number of viologen moieties (*n*) per dendrimer molecule

In the ^1^H NMR spectra (Fig. S1†), the ratio of the integration area of the aromatic region (*I*_arom_) to that of the aliphatic signals between 2.2 and 3.8 ppm (*I*_aliph_) is 3.28. The aromatic region corresponds to 8 × *n* viologen protons, and the aliphatic region corresponds to 484 dendrimer protons, as well as 11 × *n* viologen protons. Therefore, *n* is obtained from *n* = 484/((8 × *I*_aliph_/*I*_arom_) – 11). Based on *I*_aliph_/*I*_arom_ = 3.28, each dendrimer bears on average 31.8 viologen groups which correspond to a conversion yield of 99%.

### Protein purification

*Dv*MF [NiFe] hydrogenase was purified as described previously.^37^ The *petH* gene coding for the FNR of *Nostoc* sp. PCC7120 was amplified from genomic DNA excluding the 5′-end which covers 138aa of the cpcD like domain. The PCR product was cloned into the pASK-IBA7 expression vector (IBA GmbH) lacking the Strep tag II sequence. Wild type FNR proteins of *Nostoc* sp. PCC 7120 were overexpressed in 3 liter LB medium (Lennox) using *E. coli* strain BL21(DE3) as a host. Expression was induced upon attaining an OD_600_ of 0.6 by the addition of 200 mg anhydrotetracycline per liter host culture. After further cultivation for 4–5 h the cells were harvested by centrifugation and resuspended in 100 mM Tris–HCl pH 8, 10% glycerol. Cell disruption was achieved by passing twice through a French pressure cell press (1000 psig). Cell debris was separated from soluble proteins by ultracentrifugation (45 000 rpm, 1 h, 4 °C) and the supernatant subsequently cleaned from residual impurities by batch incubation treatment with 1 g DEAE-cellulose (Whatman DE52) and two filtration steps (folded filter 2V Whatman, Filtropur S 0.2 μM). The clear filtrate was supplemented by ammonium sulfate ((NH_4_)_2_SO_4_) to 40% saturation and a first fraction of contaminating soluble proteins separated from the target protein by centrifugation (10 000 rpm, 10 min, 4 °C). Further (NH_4_)_2_SO_4_ was added to the supernatant up to 70% saturation. After centrifugation (see above) the yellowish precipitate containing the FNR was resuspended in buffer 2 (5 mL, 25 mM Tris–HCl, pH 7.5) and dialyzed overnight against the same buffer. During the following chromatography steps fractions containing the FNR were easily identified by their deep yellow coloring.

Anion exchange chromatography on a DEAE Sepharose (CL-6B, Sigma) column (1.5 × 20 cm) was performed using a 50 mM step gradient of NaCl (50–500 mM) in buffer 2, yielding the FNR within the 200 mM fraction. The eluate was supplemented with (NH_4_)_2_SO_4_ up to 30% saturation prior to hydrophobic interaction chromatography done on a phenyl sepharose column (Fast Flow, Sigma). The protein is recovered between 20 and 17% in a 3% ammonium sulfate step gradient of (32–2% saturation). Yellow fractions were pooled, dialyzed against buffer 2 and concentrated up to 1 mM FNR. Target protein purity was verified by SDS PAGE and Coomassie staining.

### Fluorescence characterization

Fluorescence measurements were performed on a Leica Microsystems TCS SP8 CARS laser scanning microscope in a fully confocal optical setup. An argon gas laser excited the samples at 488 nm through a HC PL Fluotar 10x/0.3 dry objective while the emission was detected in EPI direction with photomultipliers roughly between 500 and 650 nm. Each measurement consisted of 512 or 1024 pixels in both *x* and *y* directions and up to 38 stacks in *z* direction, with pixel sizes of down to 1 μm and stack heights of 4 μm. The extension of the measurements into the third dimension allows qualitative assessment of the thickness of the sample and its (sub-) surface structure (*e.g.* the coffee ring effect or aggregates). Up to 25 (5 × 5) measurements were combined to one mosaic to show the full extent of the samples. At the end all *z*-planes were projected (maximum intensity projection) onto a single plane to allow a two dimensional comparison of the mosaics.

### AFM characterization

The AFM measurements were conducted in the AC mode by NanoWizard 3 (JPK) with cantilever of the type NSC15 (MikroMarsch). The details of film assembly are provided in the related figure legends.

### Electrochemical and size characterization

All electrochemical measurements were performed at room temperature through a Gamry Potentiostat and an Autolab PGSTAT12 Bipotentiostat. A platinum wire and Ag/AgCl/3 M KCl were used as the counter electrode and reference electrode, respectively. The details of electrode modification are provided in the related figure legends. DLS was applied to determine the size and size distribution *via* a Malvern Zetasizer Nano ZS.

## Author contributions

N. P. conceived the research. H. L performed or contributed to all electrochemical, microscopy and DLS experiments. D. B performed the electrochemical simulations. R. W. performed the synthesis of the viologen precursors, S. S. performed the synthesis and characterization of the polymer. J. H. designed, synthesized and characterized the dendrimer. K. M. contributed to confocal fluorescence experiments. E. F. conceived and performed the confocal fluorescence experiments. W. L. contributed with the hydrogenase, and M. W. and T. H. with the FNR. H. L. and N. P. wrote the manuscript. All authors commented on the manuscript.

## Conflicts of interest

There are no conflicts to declare.

## Supplementary Material

Supplementary information

## References

[cit1] Lim J. A., Lee W. H., Lee H. S., Lee J. H., Park Y. D., Cho K. (2008). Adv. Funct. Mater..

[cit2] Larson R. G. (2017). Nature.

[cit3] Deegan R. D., Bakajin O., Dupont T. F., Huber G., Nagel S. R., Witten T. A. (1997). Nature.

[cit4] Kuang M., Wang L., Song Y. (2014). Adv. Mater..

[cit5] Shen X., Ho C.-M., Wong T.-S. (2010). J. Phys. Chem. B.

[cit6] Eral H. B., Augustine D. M., Duits M. H. G., Mugele F. (2011). Soft Matter.

[cit7] Crivoi A., Duan F. (2013). Phys. Rev. E: Stat., Nonlinear, Soft Matter Phys..

[cit8] Kim H., Boulogne F., Um E., Jacobi I., Button E., Stone H. A. (2016). Phys. Rev. Lett..

[cit9] Yunker P. J., Still T., Lohr M. A., Yodh A. G. (2011). Nature.

[cit10] Davidson Z. S., Huang Y., Gross A., Martinez A., Still T., Zhou C., Collings P. J., Kamien R. D., Yodh A. G. (2017). Nat. Commun..

[cit11] Li P., Li Y., Zhou Z.-K., Tang S., Yu X.-F., Xiao S., Wu Z., Xiao Q., Zhao Y., Wang H., Chu P. K. (2016). Adv. Mater..

[cit12] Cui L., Zhang J., Zhang X., Huang L., Wang Z., Li Y., Gao H., Zhu S., Wang T., Yang B. (2012). ACS Appl. Mater. Interfaces.

[cit13] Talbot E. L., Yang L., Berson A., Bain C. D. (2014). ACS Appl. Mater. Interfaces.

[cit14] Accardo J. V., Kalow J. A. (2018). Chem. Sci..

[cit15] Bae S.-H., Zhao H., Hsieh Y.-T., Zuo L., Nicholas D. M., Rim Y. S., Li G., Yang Y. (2016). Chem.

[cit16] Wang Z. J., Ghasimi S., Landfester K., Zhang K. A. I. (2015). Adv. Mater..

[cit17] Nocera D. G. (2012). Acc. Chem. Res..

[cit18] Heller A., Feldman B. (2008). Chem. Rev..

[cit19] Plumeré N., Rudiger O., Oughli A. A., Williams R., Vivekananthan J., Poller S., Schuhmann W., Lubitz W. (2014). Nat. Chem..

[cit20] Reuillard B., Warnan J., Leung J. J., Wakerley D. W., Reisner E. (2016). Angew. Chem., Int. Ed..

[cit21] Fourmond V., Stapf S., Li H., Buesen D., Birrell J., Rudiger O., Lubitz W., Schuhmann W., Plumeré N., Léger C. (2015). J. Am. Chem. Soc..

[cit22] Bartlett P. N., Pratt K. F. E. (1995). J. Electroanal. Chem..

[cit23] Andrieux C. P., Dumas-Bouchiat J. M., Savéant J. M. (1980). J. Electroanal. Chem. Interfacial Electrochem..

[cit24] Andrieux C. P., Dumas-Bouchiat J. M., Savéant J. M. (1982). J. Electroanal. Chem. Interfacial Electrochem..

[cit25] Blauch D. N., Savéant J. M. (1992). J. Am. Chem. Soc..

[cit26] Noh Y.-Y., Lee C.-L., Kim J.-J., Yase K. (2003). J. Chem. Phys..

[cit27] Kothe T., Pöller S., Zhao F., Fortgang P., Rögner M., Schuhmann W., Plumeré N. (2014). Chem. - Eur. J..

[cit28] Oughli A. A., Conzuelo F., Winkler M., Happe T., Lubitz W., Schuhmann W., Rüdiger O., Plumeré N. (2015). Angew. Chem., Int. Ed..

[cit29] Bracher P. J., Snyder P. W., Bohall B. R., Whitesides G. M. (2011). Origins Life Evol. Biospheres.

[cit30] Massey J., Power K. N., Manners I., Winnik M. A. (1998). J. Am. Chem. Soc..

[cit31] Esfand R. and Tomalia D. A., Laboratory synthesis of poly(amidoamine)(PAMAM) dendrimers, John Wiley, Chichester, 2001. 10.1016/s1359-6446(01)01757-3 11301287

[cit32] Buesen D., Li H. and Plumeré N., 2018, to be submitted.

[cit33] Chen L., Evans J. R. G. (2010). J. Colloid Interface Sci..

[cit34] Hampton M. A., Nguyen T. A. H., Nguyen A. V., Xu Z. P., Huang L., Rudolph V. (2012). J. Colloid Interface Sci..

[cit35] Heath G. R., Li M., Rong H., Radu V., Frielingsdorf S., Lenz O., Butt J. N., Jeuken L. J. C. (2017). Adv. Funct. Mater..

[cit36] Abou Hamdan A., Burlat B., Gutiérrez-Sanz O., Liebgott P.-P., Baffert C., de Lacey A. L., Rousset M., Guigliarelli B., Léger C., Dementin S. (2013). Nat. Chem. Biol..

[cit37] Fichtner C., Laurich C., Bothe E., Lubitz W. (2006). Biochemistry.

