# Supplementary material for: Preventing the coffee-ring effect and aggregate sedimentation by in situ gelation of monodisperse materials
Source: Chem Sci. 2018 Aug 23;9(39):7596–605. doi: 10.1039/c8sc03302a (PMC6182772; doi:10.1039/c8sc03302a)
Supplement: Supplementary file 1 [file SC-009-C8SC03302A-s001.pdf]

Electronic Supplementary Material (ESI) for Chemical Science.
This journal is © The Royal Society of Chemistry 2018

Supporting Information

Title: Preventing the Coffee-Ring Effect and Aggregate Sedimentation by in-Situ Gelation of Monodisperse Materials

Huaiguang Li[a], Darren Buesen[a], Rhodri Williams[a], Joerg Henig[a], Stefanie Stapf[a], Kallol Mukherjee[b], Erik Freier[c], Wolfgang Lubitz[d], Martin Winkler[e], Thomas Happe[e] and Nicolas Plumeré[a]*

[a] a. Center for Electrochemical Sciences (CES), Faculty of Chemistry and Biochemistry, Ruhr University Bochum, Universitätsstr. 150, D-44780 Bochum, [b] Lehrstuhl für Physikalische Chemie II, Ruhr-Universität Bochum, 44780 Bochum, Germany, [c] Leibniz-Institut für Analytische Wissenschaften -ISAS- e.V., 44227 Dortmund, Germany, [d] Max-Planck-Institut für Chemische Energiekonversion, Stiftstrasse 34-36, 45470 Mülheim an der Ruhr, Germany, [e] Lehrstuhl Biochemie der Pflanzen, Ruhr-Universität Bochum, 44780 Bochum, Germany.

* Corresponding author
E-mail: nicolas.plumere@rub.de

Table of Contents:

Scheme S1: Structure of third generation poly(amido amine) (PAMAM G3) dendrimer with 32 thiol functionalized viologen terminal groups.

Scheme S2: Synthesis of the third generation poly(amido amine) (PAMAM G3) dendrimer with thioacetate functionalized viologen terminal groups.

Figure S1: ^{1}H NMR spectrum of the thioacetate protected viologen-modified PAMAM G3 dendrimer in D_2O.

Figure S2: UV-Visible spectra of viologen-modified polymer and dendrimer.

Figure S3: Thickness of dendrimer films determined via AFM.

Figure S4: Film roughness determination from the electrochemical method.

Figure S5: Morphology of the hydrogel films assembled by drop-casting on gold and carbon surfaces.

Figure S6: Determination of the film thickness distribution for a dendrimer film prepared on a gold substrate with a horizontal orientation (0°).

Figure S7: Determination of the film thickness distribution for a dendrimer film prepared on a gold substrate with a vertical orientation (90°).

Figure S8: Determination of the film thickness distribution for a dendrimer film prepared on a gold substrates with an upside down orientation (180°).

Figure S9: IDA measurements of viologen-modified dendrimer films.

Table S1: Charge obtained from integration of the area.

Figure S10: Catalytic current densities for NADPH oxidation with various film thicknesses.

Figure S11: Catalytic current density for NADPH oxidation.

Figure S12: Linear film thickness dependency on the amount of drop-cast dendrimer.

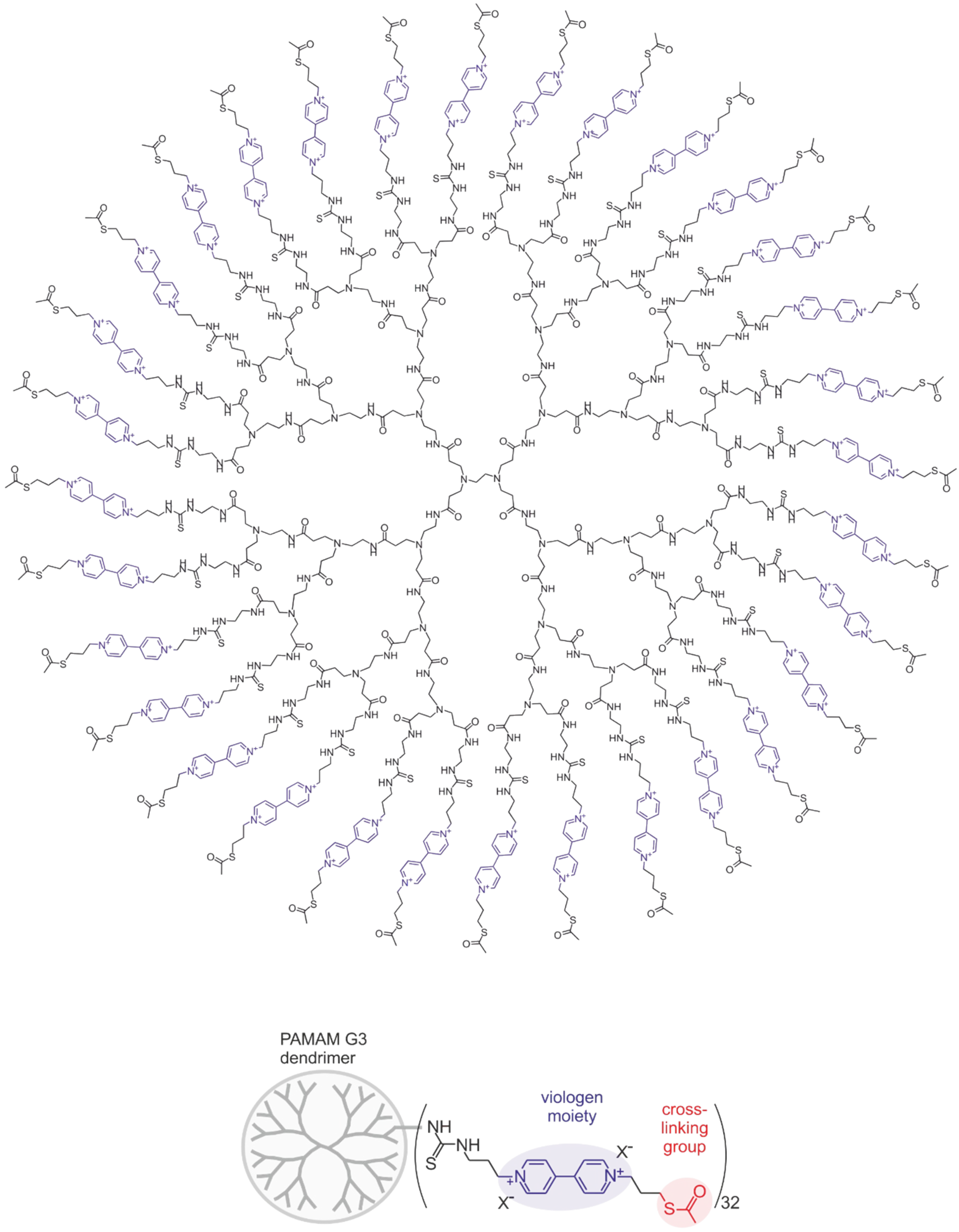

2

Scheme S1: Structure of third generation poly(amido amine) (PAMAM G3) dendrimer with 32 thiol functionalized viologen terminal groups (**2**).

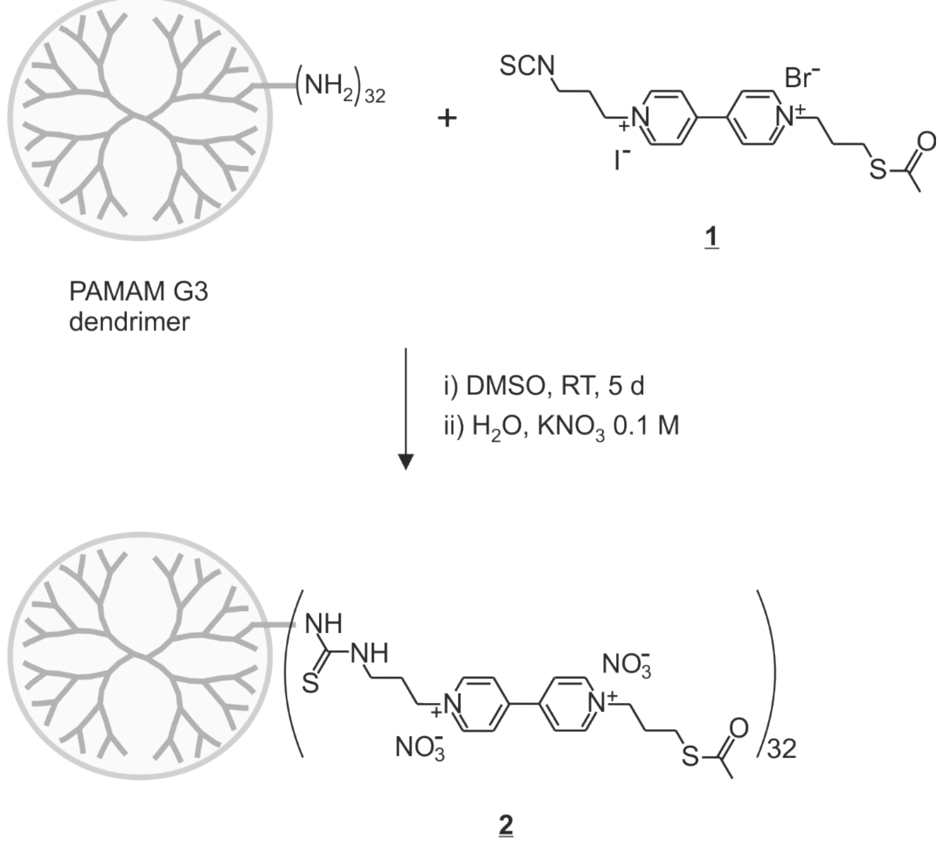

Scheme S2: Synthesis of the third generation poly(amido amine) (PAMAM G3) dendrimer with thioacetate functionalized viologen terminal groups (**2**), based on the reaction of the amino head group of commercially available PAMAM G3 dendrimers with a viologen monomer terminated by both isothiocyanate and thioacetate groups (1-(3-(acetylthio)propyl)-1'-(3-isothiocyanatopropyl)-[4,4'-bipyridine]-1,1'-diium iodide bromide, **1**). The synthesis and full characterization of the viologen derivative **1** was reported previously.[1]

Quantification of the film thickness distribution from the electroanalytical method. [2]

The electroanalytical method developed and used for the characterization of redox film preparations is based on analysis of the peak currents obtained from the Linear Sweep Voltammograms (LSV). The model is based on a Langmuir assumption (no interaction of neighboring redox active mediator molecules). However, the LSVs obtained in our experimental system (i.e. Figure S4B) include contributions related to interactions between the redox mediator molecules, according to the Frumkin isotherm. In this case, the model is still applicable, but requires conversion of the peak currents to their Langmuir equivalents before further data processing.

Frumkin isotherm correction. In the case of the viologen-based dendrimer, the interaction between the redox centers requires the use of the Frumkin isotherm[3] to describe the current response in the surface confined case. **Equation S1** includes a correction factor (ψ_p) based on the Frumkin isotherm:

$$i_p = \frac{n^2F^2}{RT}vA\Gamma_T\psi_p$$

(Equation S1)

with

$$\psi_p = \frac{1}{4 - 2\upsilon G\theta_T}$$

Where *n, F, R, T, A* and ν are, respectively, the number of electron transferred, the Faraday constant, the gas constant, temperature, the electrode surface area and the scan rate; $G = a_O + a_R - 2a_{OR}$ (a_{OR}, a_O and a_R are the R-O, O-O, and R-R interaction parameters. υ is the number of water molecules displaced.); and $\theta_T = \theta_O + \theta_R$ (θ_O and θ_R are the fractional coverage of O and R respectively. $\theta_O = \Gamma_O / \Gamma_m$ and $\theta_R = \Gamma_R / \Gamma_m$ with Γ_m the maximal value for Γ_O and Γ_R).

The value of ψ_p can be easily derived from **Equation S1** based on the slope of a plot of i_p versus ν with known parameters of *n, F, R, T, A* and Γ_T for CVs in the surface confined case (the surface coverage of Γ_T is calculated by integration of the charge). For the viologen-based dendrimer on Au electrode, the value of the factor $1/\psi_p$ is 2.06.

In the next step, with the use of the other relevant experimental parameters (such as concentration, electron diffusion coefficient, etc.), the peak currents (y-axis) and the scan rates

(x-axis) are normalized by application of the expressions shown in the respective axes (i.e. Figure S4C,D). In the expression for peak current normalization, i_p is the current density.

Next, the value of the dimensionless current at the reference line (where the value of the dimensionless current is equal to 2) is read from the dimensionless plot. Using this value, and a correlation plot, the information related to the film thickness distribution is directly obtained (Weibull Distribution shape factor). This value is then used to generate a plot of the probability distribution function (normalized histogram) for the underlying film thickness (i.e Figure S4E,F).

In order to visualize what the resulting surface could possibly look like, for comparison with AFM or optical microscope images, the probability distribution function is transformed into a three-dimensional representation (i.e. Figure S4G,H). When comparing the 3D representation from the electrochemical method with images from AFM, care must be taken to regard the limiting and complementary nature of the information provided by these two methods. For microscopy type methods such as AFM, for example, the obtained images are highly dependent on the sampling area, which is very small when compared to the entire film. For the electrochemical method, although the entire film is represented, the number of points used to represent the film is arbitrarily chosen (in Figure S4G,H this number is 100), and the locations of the film sections are also arbitrarily chosen, since this information is not accessible using the electrochemical method. Therefore, the electrochemical and AFM methods provide complementary information. When the results of these methods are in good general agreement, a high degree of assurance regarding the underlying thickness distribution and roughness of the film preparation is obtained.

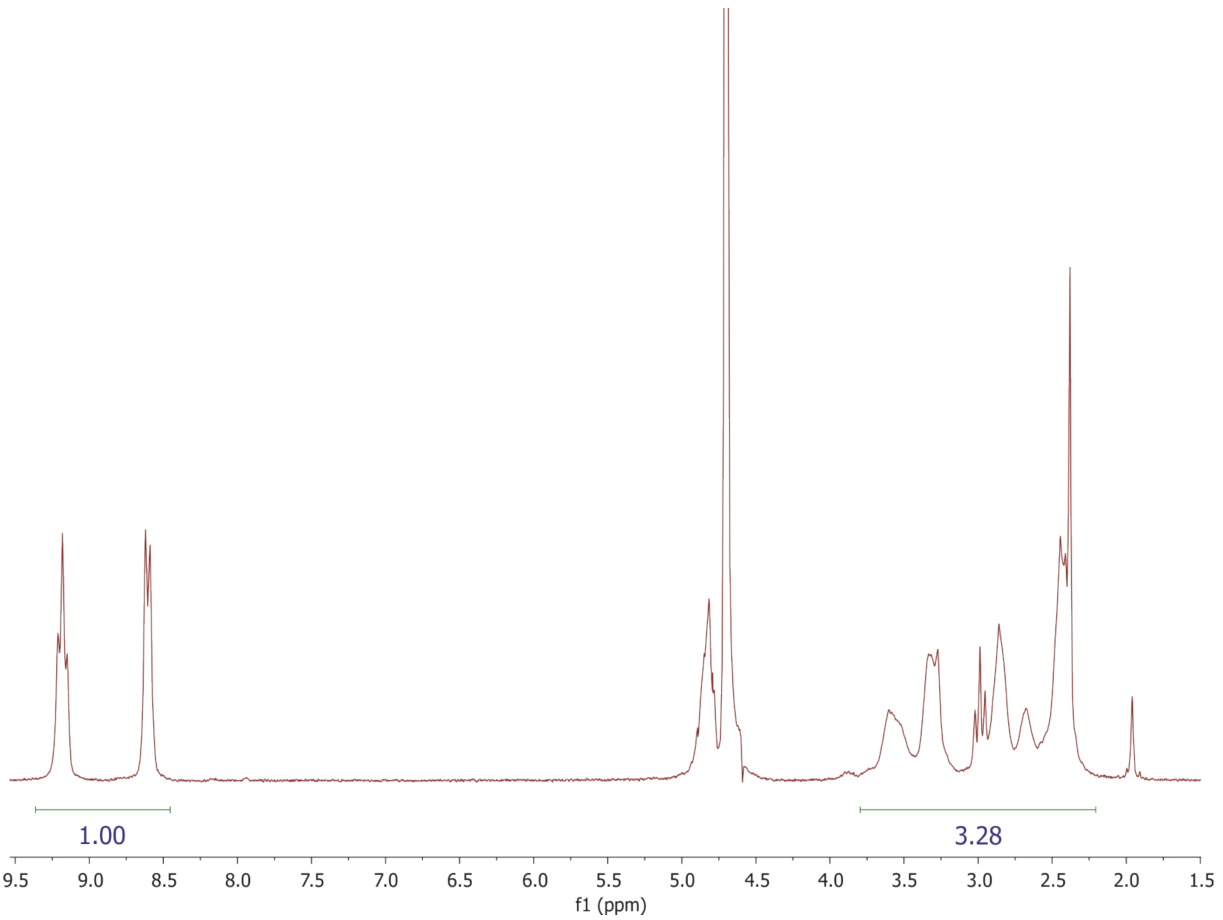

Figure S1: ^{1}H NMR spectrum of the thioacetate protected viologen-modified PAMAM G3 dendrimer in D_2O. The signals between 8.5 and 9.5 ppm are assigned to the protons of the aromatic ring of the viologens while the signals between 2.2 and 3.8 ppm are attributed to the protons of the dendrimer core and the alkyl chains of the viologen moieties (Yield: 99%).

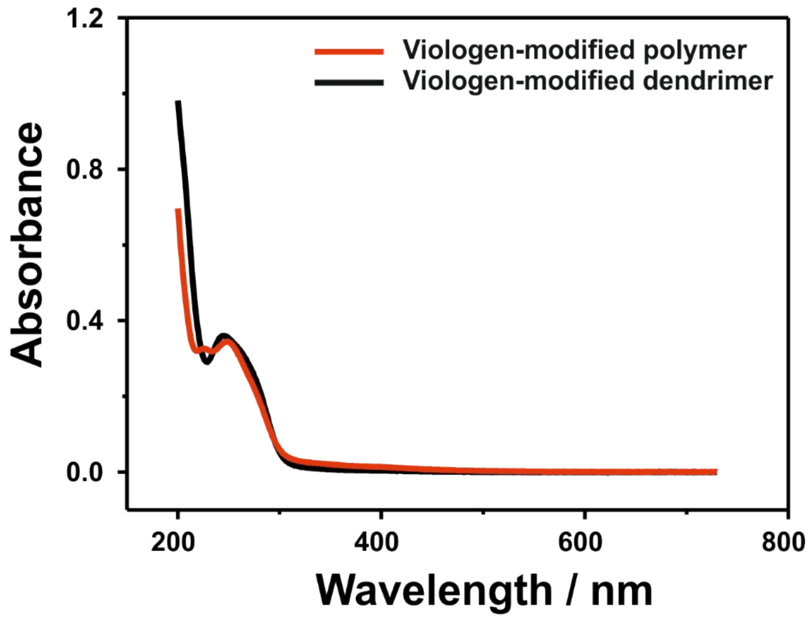

Figure S2: UV-Visible spectra of viologen-modified polymer (2 ml aqueous suspension solution, 0.014 mg mL^{-1}) and of viologen-modified dendrimer (2 ml aqueous suspension solution, 0.011 mg mL^{-1}).

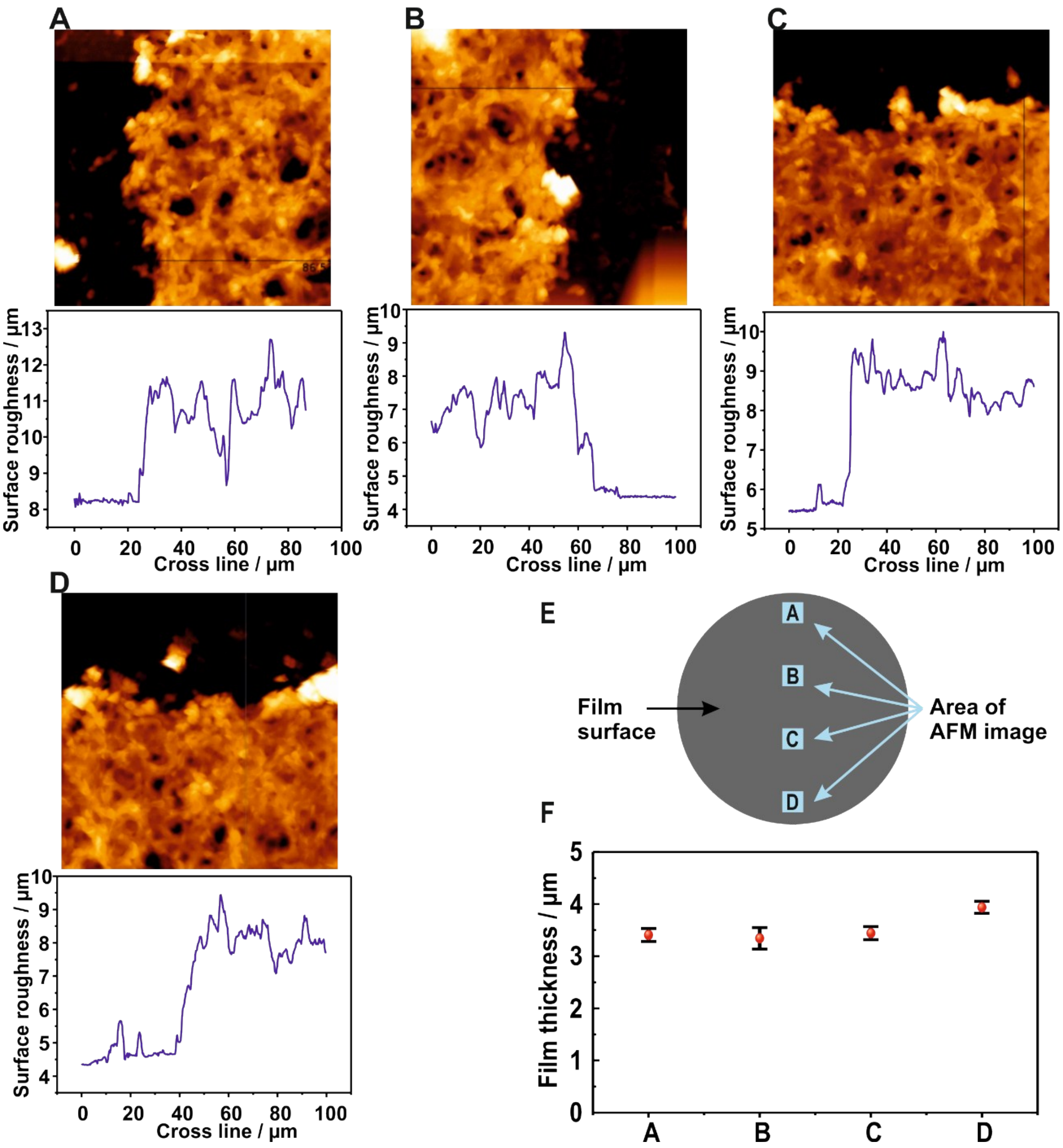

Figure S3: Thickness of dendrimer films determined via AFM. A) - **D**) AFM measurements in different areas of the electrode surface, after surface scratching for local removal of the dendrimer film. The film was assembled from $6.8 \cdot 10^{-10}$ mol viologen-modified dendrimer (2 µL, 0.34 mM) drop-cast onto a Au electrode. **E**) Sketch of the areas on the electrode investigated by AFM, corresponding to the image A) to D). **F**) Film thicknesses extracted from the four AFM images. The standard deviations correspond to the surface roughness of the dendrimer films extracted from the AFM images. The AFM images were recorded in AC mode on a 50 µm × 50 µm area. Before the measurement, the film was scratched with a scalpel to reveal the underlying electrode material and thus enable film thickness determination.

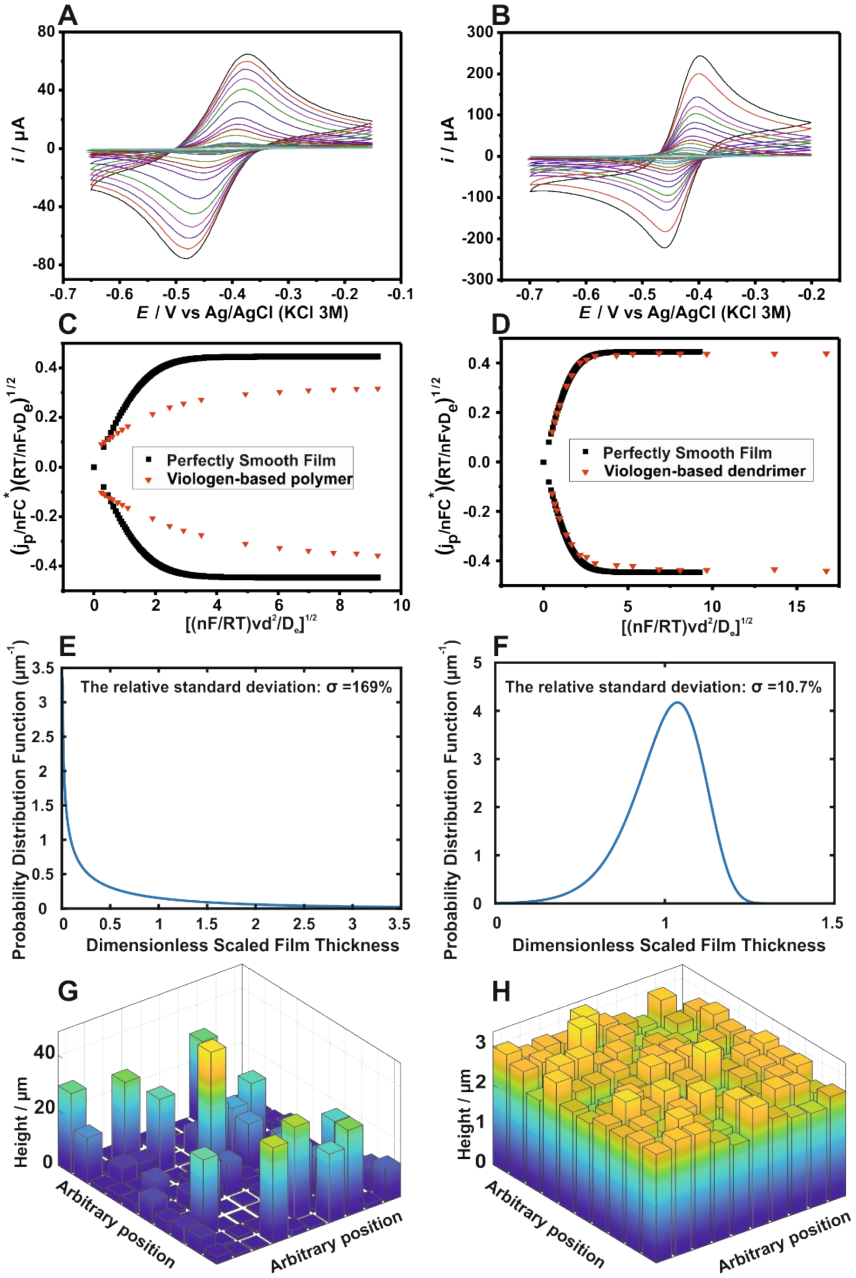

Figure S4: Film roughness determination from the electrochemical method. Cyclic voltammograms (CVs) of **A**) viologen-modified polymer film and **B**) viologen-modified dendrimer films at different scan rates on glassy carbon electrodes. Variation of the dimensionless peak currents with dimensionless scan-rate $[(nF/RT)vd^2/D_e)]^{1/2}$ for **C**) viologen-modified polymer film and for **D**) viologen-modified dendrimer films. In both cases, the experimental data (red triangles) are compared to the theoretical behavior for a perfectly homogeneous film (black squares). The parameters used for non-dimensionalization of the experimental data are $D_e = 1.15 \cdot 10^{-8}$ cm^2 s^{-1} (the corresponding measurements are in the Figure S9), of $C = 3.03 \cdot 10^{-4}$ mol cm^{-3}, d = 2.6 µm, and $1/\psi_p$ = 1.48 for viologen-modified dendrimer, and $D_e = 4.7 \cdot 10^{-9}$ cm^2 s^{-1}, C = 1.09 · 10^{-4} mol cm^{-3}, and d = 7.32 µm for the viologen-modified polymer. Probability distribution function of **E**) viologen-modified polymer and **F**) viologen-modified dendrimer and 3D representations of the surfaces of films from **G**) viologen-modified polymer and **H**) viologen-modified dendrimer according to the electrochemical method for film thickness distribution determination. The measurements were performed in phosphate buffer (100 mM, pH 7.2) under anaerobic conditions.

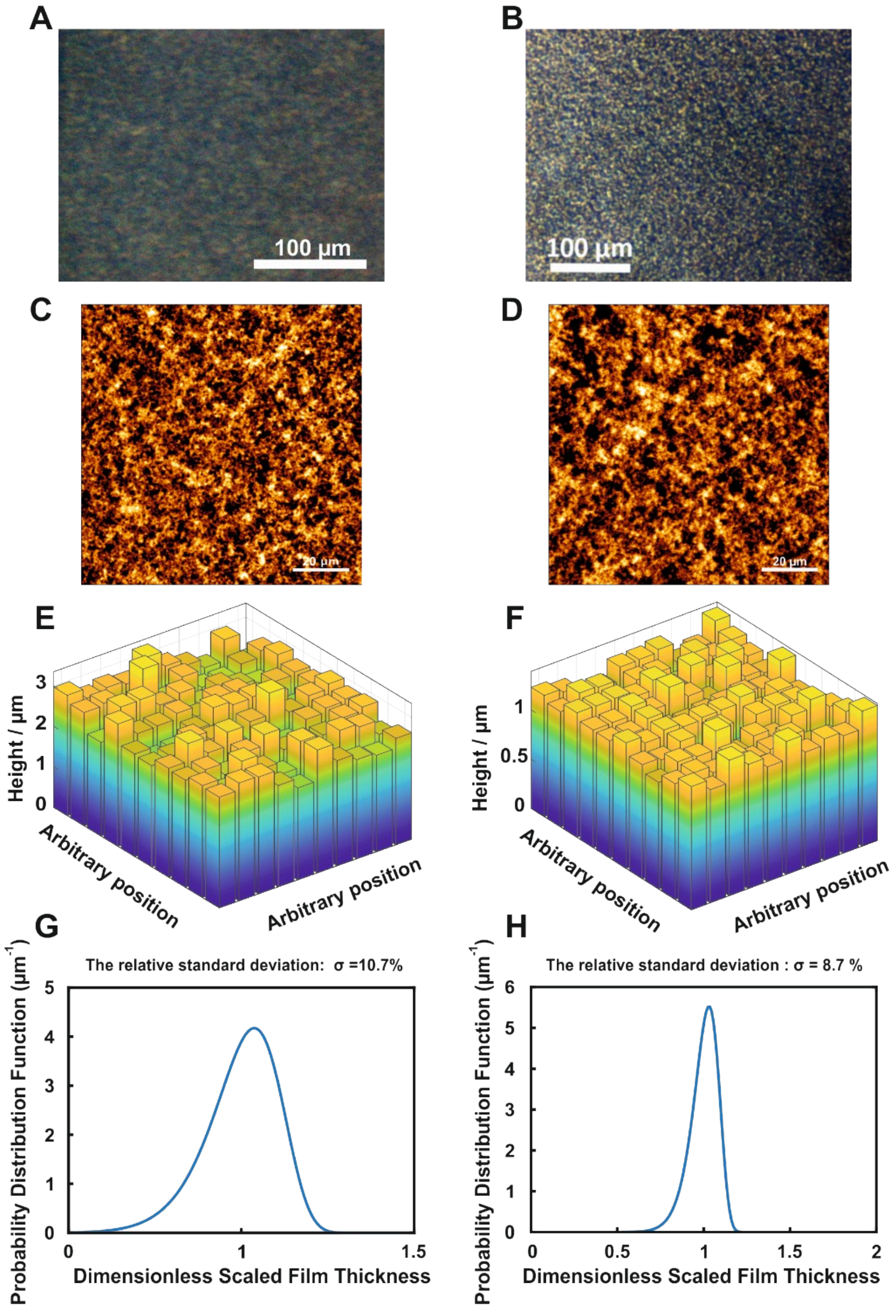

Figure S5: Morphology of the hydrogel films assembled by drop-casting on carbon and Au surfaces: viologen-modified dendrimer (**A**, **C**, **E** and **G**) on glassy carbon electrodes (3 mm in diameter) and (**B**, **D**, **F** and **H**) on Au electrodes (2 mm in diameter). **A**) and **B**) Optical microscopy images. **C**) and **D**) AFM micrographs showing the surface morphology over 100 µm × 100 µm areas. **E**) and **F**) 3D representations of the film thickness distributions extracted from the electrochemical method. **G**) and **H**) Probability distribution function. The disk electrodes were modified with a surface coverage of 200 µg cm^{-2}, and the gelation process was carried out with a total dendrimer suspension volume of 2.5 µL, including 0.5 µL of Tris buffer solution (100 mM, pH 9.0) at RT under a water saturated atmosphere.

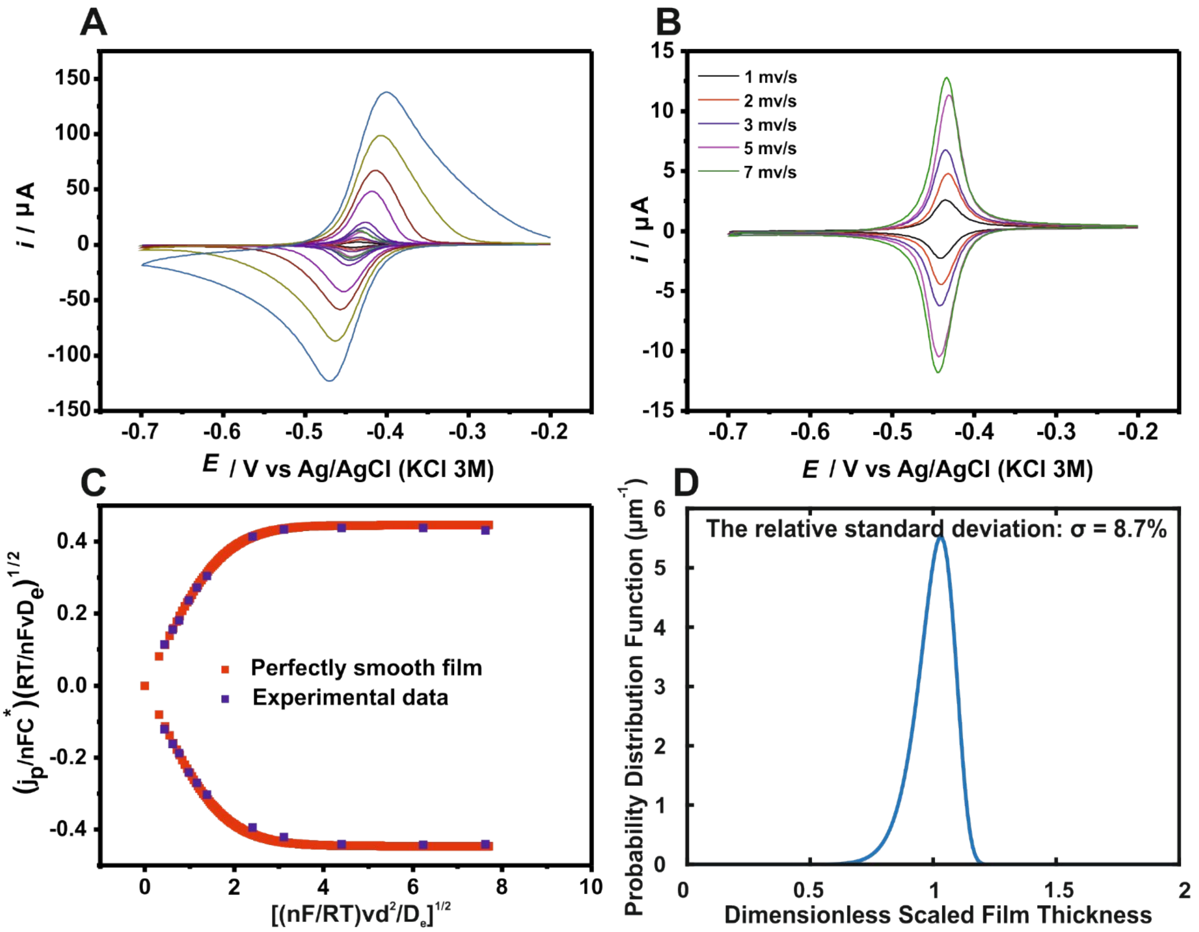

Figure S6: Determination of the film thickness distribution for a dendrimer film prepared on a gold substrate with a horizontal orientation (0°) using the electrochemical method A) CVs of viologen-modified dendrimer films at different scan rates **B**) CVs at slow scan rate from A). **C**) Variation of dimensionless peak currents with dimensionless scan-rate $[(nF/RT)vd^2/D_e)]^{1/2}$: Theoretical dimensionless current for a perfectly smooth film (red squares) and for the experimental peak currents extracted from (A) (blue squares). The parameters used for the non-dimensionalization of the experimental data are the same as those used in the caption of Figure S4. **D**) Probability distribution function. The diameter of the Au electrode was 2 mm, and the measurements were performed in phosphate buffer (100 mM, pH 7.2) under anaerobic conditions.

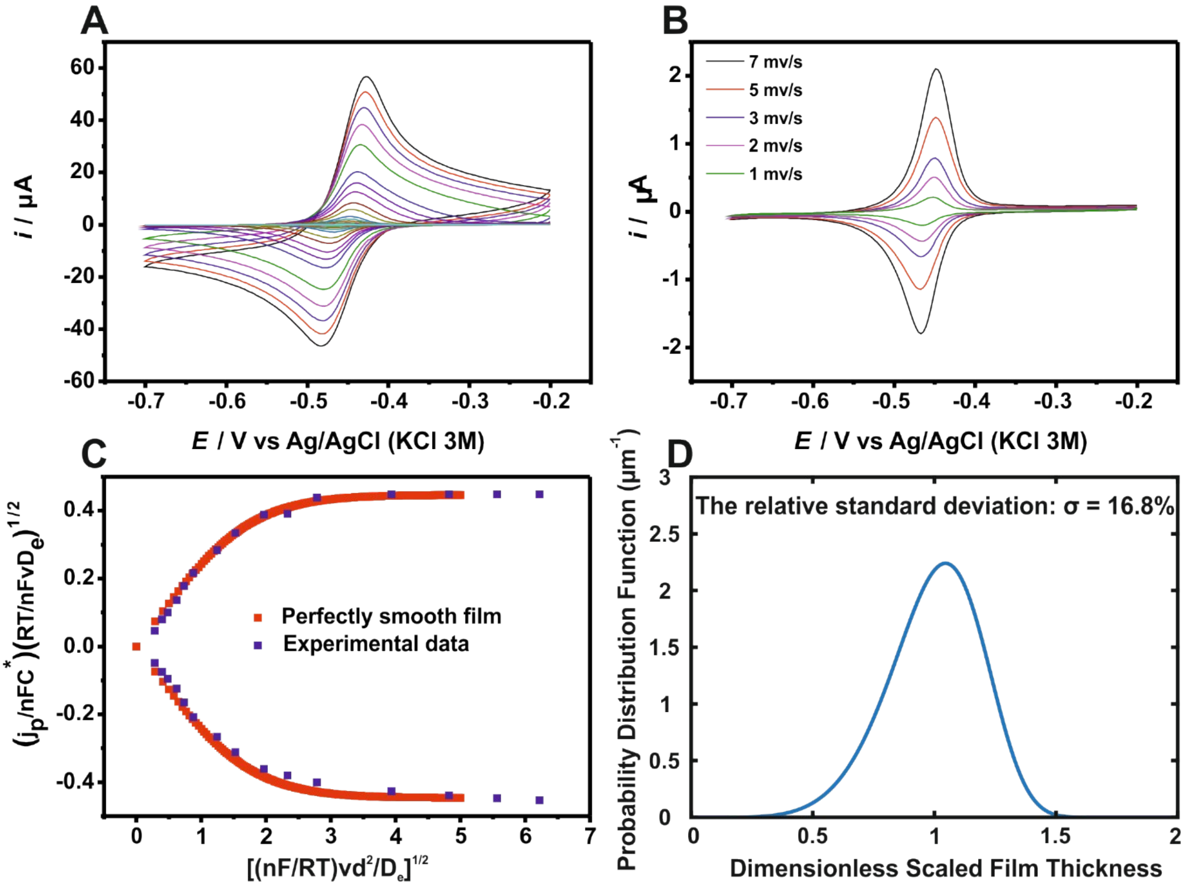

Figure S7: Determination of the film thickness distribution for a dendrimer film prepared on a gold substrate with a vertical orientation (90°) using the electrochemical method. A) CVs of viologen-modified dendrimer films at different scan rates **B**) CVs at slow scan rate from A). **C**) Variation of dimensionless peak currents with dimensionless scan-rate $[(nF/RT)vd^2/D_e)]^{1/2}$: Theoretical dimensionless current for a perfectly smooth film (red squares) and for the experimental peak currents extracted from (A) (blue squares). The parameters used for the non-dimensionalization of the experimental data are the same as those used in the caption of Figure S4. **D**) Probability distribution function. The diameter of the Au electrode was 2 mm, and the measurements were performed in phosphate buffer (100 mM, pH 7.2) under anaerobic conditions.

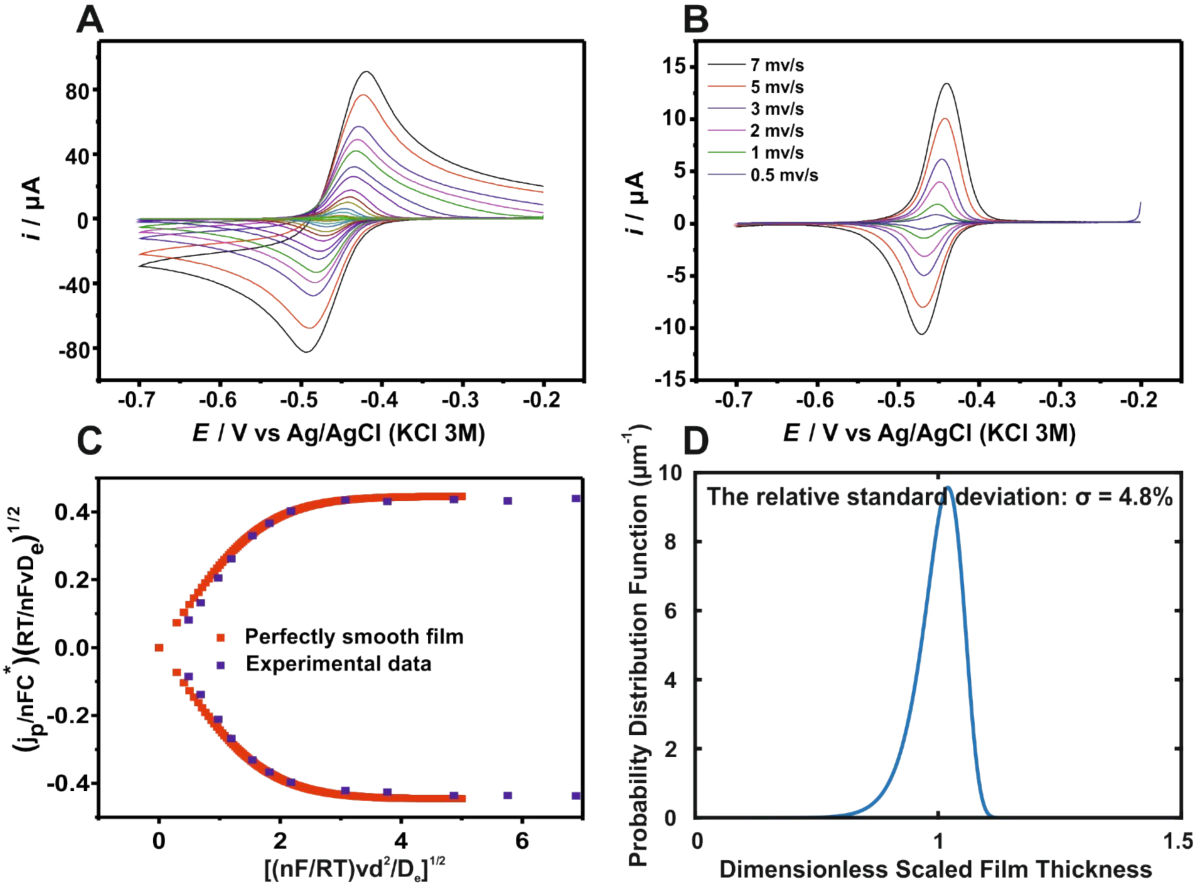

Figure S8: Determination of the film thickness distribution for a dendrimer film prepared on a gold substrates with an upside down orientation (180°) A) CVs of viologen-modified dendrimer films at different scan rates **B**) CVs at slow scan rate from A). **C**) Variation of dimensionless peak currents with dimensionless scan-rate $[(nF/RT)vd^2/D_e)]^{1/2}$: Theoretical dimensionless current for a perfectly smooth film (red squares) and for the experimental peak currents extracted from (A) (blue squares). The parameters used for the non-dimensionalization of the experimental data are the same as those used in the caption of Figure S4. **D**) Probability distribution function. The diameter of the Au electrode was 2 mm, and the measurements were performed in phosphate buffer (100 mM, pH 7.2) under anaerobic conditions.

Determination of the apparent electron diffusion coefficient (D_e) in redox active films.

Interdigitated array electrodes (IDA) were employed for the determination of D_e in viologen-modified dendrimer films. IDA electrodes allow for the calculation of D_e from the limiting current (i_L) obtained from generator-collector mode, and from the charge (Q) required for the reduction of all viologens obtained from generator-generator mode (and at slow scan rate). Then, D_e can be calculated from Q and from i_L by:

$$De = \frac{i_L * dp}{Q} \frac{N}{N-1}$$

(Equation S2)

Where N is the number of IDA fingers, d is the gap between the fingers and p is the center-to-center width of a two-finger segment.

Figure S9A displays the slow scan rate CVs obtained in generator-generator mode. The two working electrodes are shortened together and swept in a sufficiently wide potential range that ensures the complete oxidation as well as the complete reduction of the viologen moieties in the film. The CVs show a small peak separation (23 ± 2.5 mV) between the anodic and cathodic current responses that is characteristic for surface confined species. The peak currents are proportional to scan rate between 2 and 10 mV s^{-1} (**Figure S9B**), indicating complete oxidation or reduction of the viologen hydrogel in each potential sweep. Thus, the values obtained for Q that were extracted by integration of the area under the cathodic curve are associated with the one-electron interconversion between the viologen dication and the viologen radical cation (**Table S1**). The calculated charge slightly increases with decreasing scan rate due to increased inaccuracy in the background current at the lower scan rates. Therefore, the average value of these scan rates was used in the determination of D_e.

In generator-collector mode, the generator electrode was swept toward -0.8 V vs. Ag/AgCl pseudo reference electrode (RE) to reduce the oxidizing viologen, while the collector electrode was maintained at - 0.2 V to re-oxidize the reducing viologen. The formation of crossed concentration gradients of the reduced and oxidized viologen results in a steady-state current (i_L) at the collector electrode (**Figure S9C**). Andrieux *et al.* pointed out that the mobility and concentration of the counter-ion does indeed affect the transient response in the redox hydrogels.[4] However, contributions related to ion migration are avoided in steady-state measurements. Therefore, **Equation S2** is valid for the calculation of D_e from i_L and Q**.**

The average value of D_e for the dendrimer films is (1.15 ± 0.1) 10^{-8} cm^2 s^{-1}. This value was independent of the dimensions and the geometry of the IDAs. Additionally, the D_e value was independent of electrolyte concentration, demonstrating that the counter ion transfer associated with electron transfer is non-limiting. In comparison, the D_e value obtained for viologen-modified polymer films, which consist of the same viologen moieties, was (4.7 ± 1.7) 10^{-9} cm^2 s^{-1},[5] which is about 2 times lower than for the viologen-modified dendrimer hydrogel.

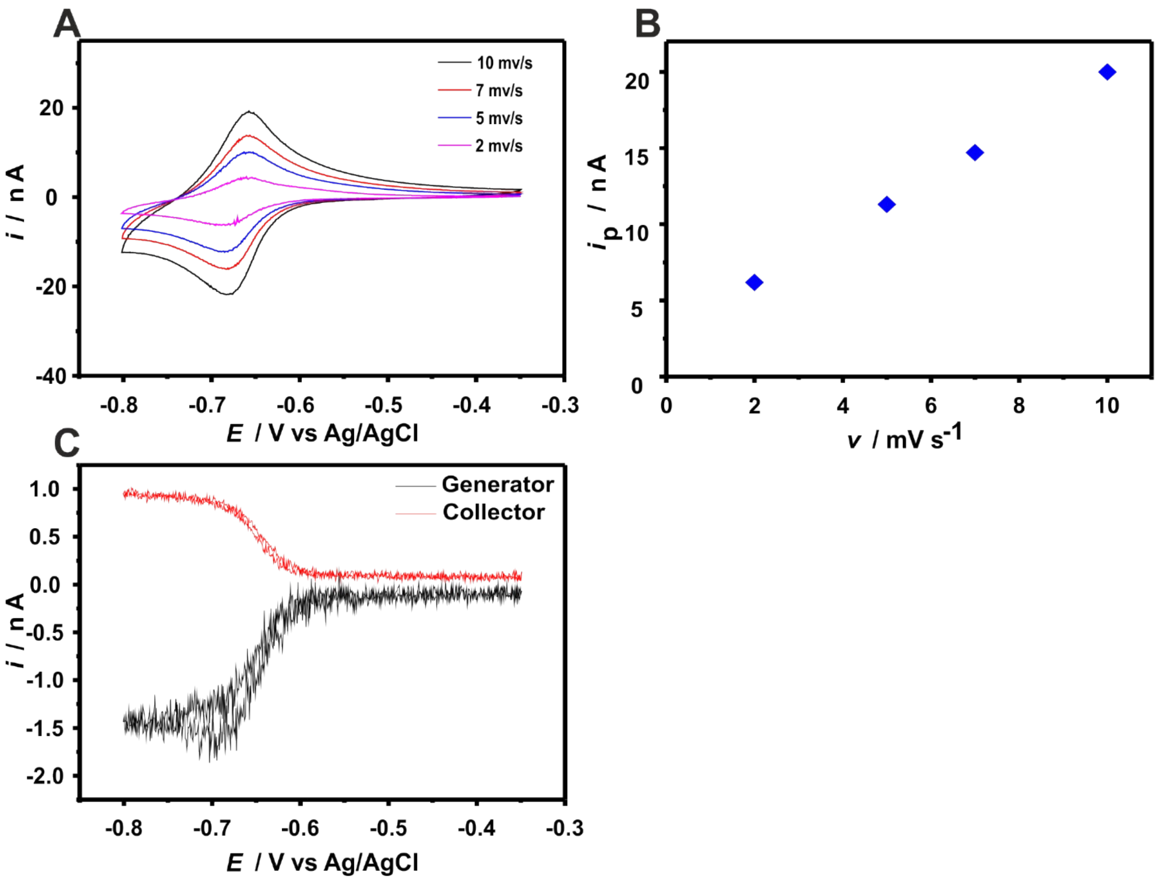

Figure S9: Measurements of the apparent diffusion coefficient of viologen-modified dendrimer films based on Interdigitated array (IDA) electrodes. A) CVs at slow scan rates (from 2 mV s^{-1} to 10 mV s^{-1}) in generator-generator mode. The applied potential (*E*) is given versus a Ag/AgCl pseudo-RE. **B**) Peak currents (i_P) with background correction vs. scan rate (*v*) derived from A) at low scan-rate. **C**) CV (0.1 mV s^{-1}) at the generator electrode and current response at the collector electrode (applied potential -0.2 V vs. Ag/AgCl pseudo RE) in generator-collector mode. The measurements were performed in phosphate buffer (100 mM, pH 7.2) under anaerobic conditions. IDA electrodes (ED-IDA6-Au, Micrux Technologies) consisted of 30 pairs of Au fingers. The width of finger and the gap between fingers are 5 μm. The pseudo-reference electrode was modified with a Ag/AgCl ink.

Table S1: Charge obtained from integration of the area under the cathodic curve of the CVs given in Figure S9A at slow scan rate.

**Scan rates**	7 mV s^{-1}	5 mV s^{-1}	2 mV s^{-1}
**Charge**	1.3 10^{-7} C	1.35 10^{-7} C	1.70 10^{-7} C

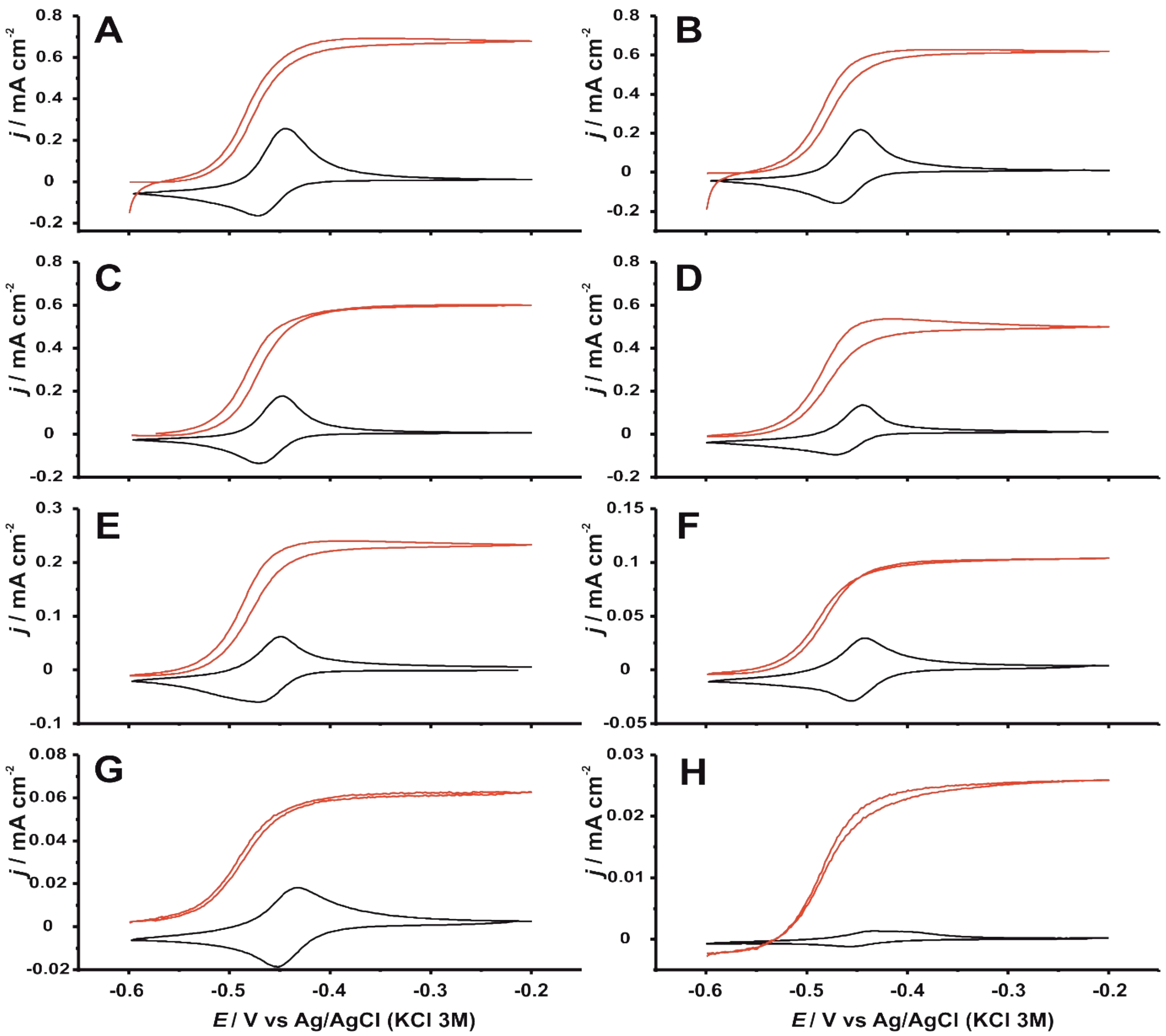

Figure S10: Catalytic current densities for NADPH oxidation with various film thicknesses. Cyclic voltammograms for Au electrodes (2 mm in diameter) drop-coated with various volumes of a viologen-modified dendrimer solution (0.34 mM) and FNR (0.1 mM) in the presence (red traces) and in the absence (black traces) of NADPH (40 mM), and at a scan rate at 2 mV s^{-1} in phosphate buffer (100 mM, pH 7.2) under anaerobic conditions. The drop-cast volumes were **A**) 4 µl, **B**) 3.5 µl, **C**) 3 µl, **D**) 2 µl, **E**) 1 µl, **F**) 0.4 µl, **G**) 0.2 µl and **H**) 0.05 µl. Additional phosphate buffer (100 mM, pH 7.0) was added to each preparation so that total volume would be 4µl.

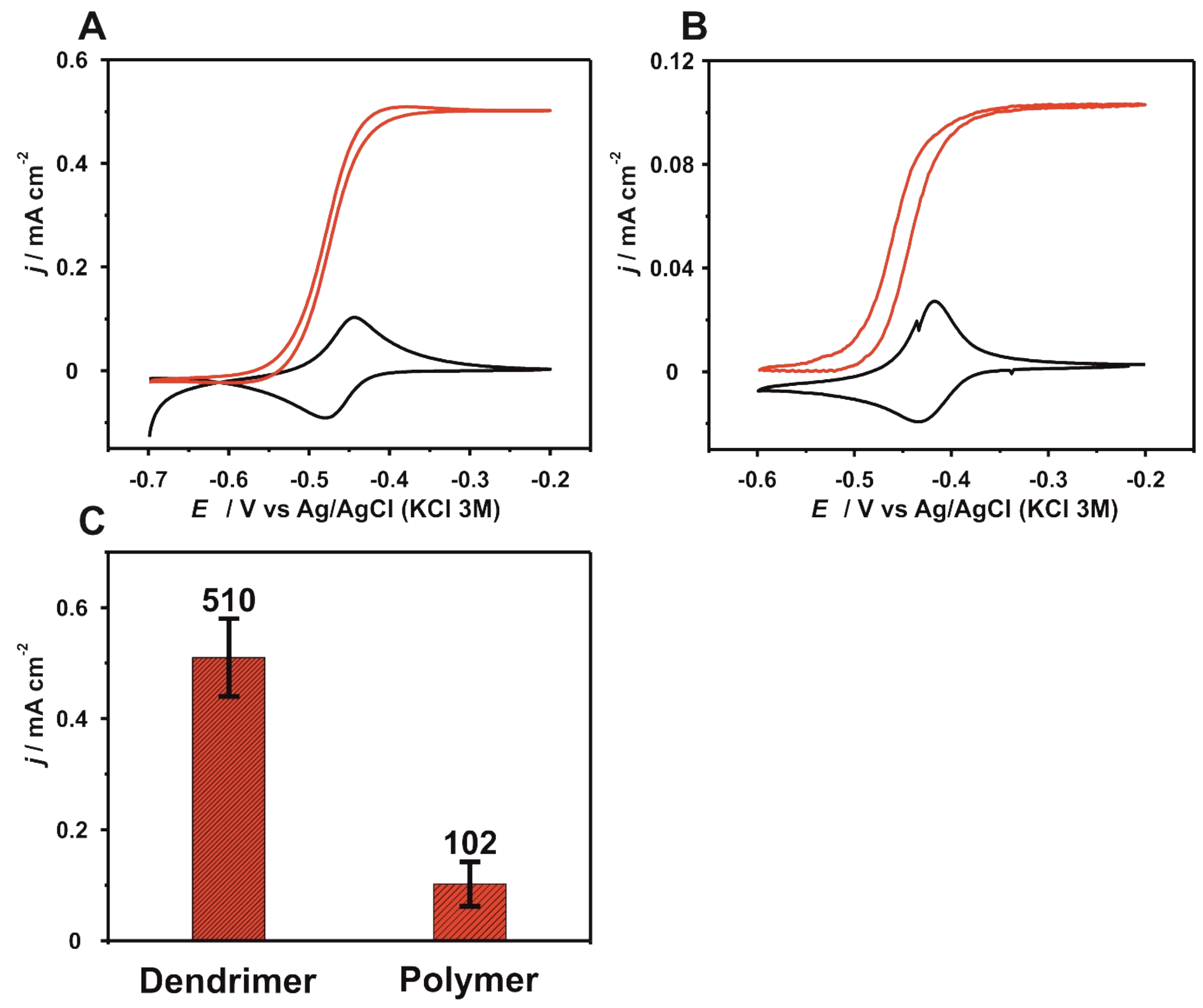

Figure S11: Catalytic current density for NADPH oxidation. Cyclic voltammograms for FNR immobilized in films of **A**) viologen-modified dendrimer and **B**) viologen-modified polymer in presence (red traces) and absence (black trace) of NADPH. **C**) Comparison of catalytic current densities of FNR immobilized in viologen-modified dendrimer and in viologen-modified polymer films respectively. The electrodes were prepared by drop-casting 2 µL of a viologen-modified dendrimer mixture (1µL, 7.14 mg/mL) or viologen-modified polymer mixture (1µL, 9.55 mg/mL) with FNR (1 µL, 0.1 mM). The standard deviations used in the error bars are obtained from two replicates from two individual preparations under the same conditions.

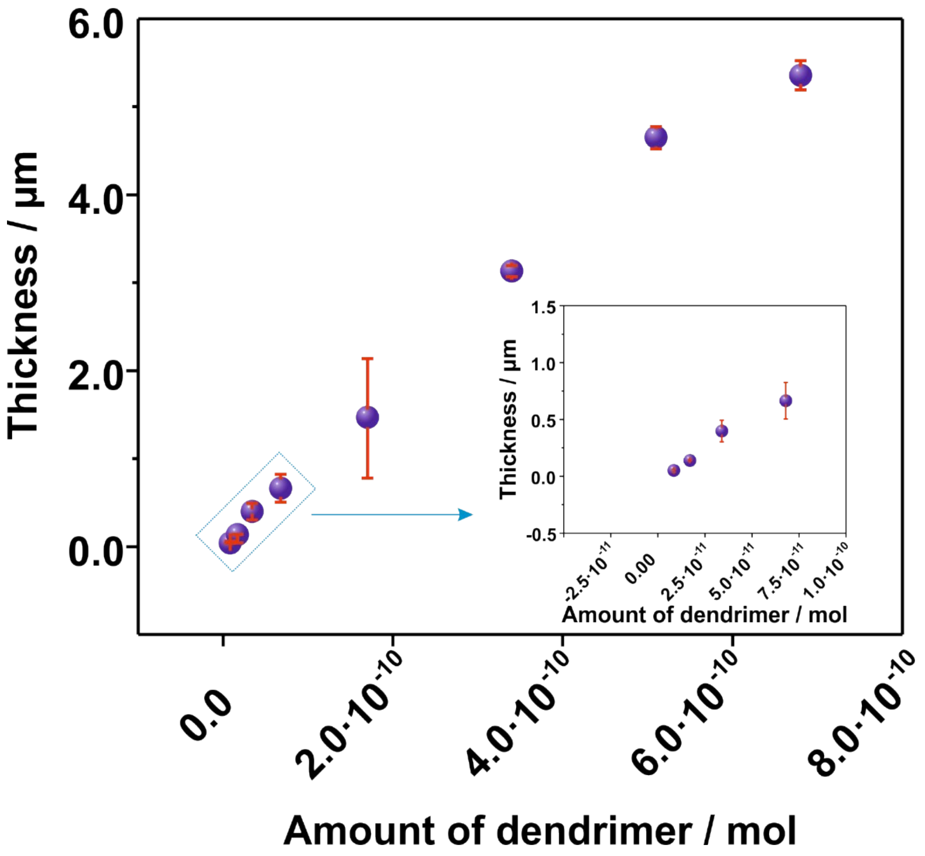

Figure S12: Linear film thickness dependency on the amount of drop-cast dendrimer. The film thicknesses (l) were obtained from the viologen surface coverage (Γ) and from the viologen concentration (C) according to $l = \Gamma / C$.[5] The values of Γ and C were, respectively, extracted from the integration of the CV wave at slow scan rates and from the peak currents of the CVs at fast scan rates according to the Randles Sevcik equation and using the value of D_e ($1.15 \cdot 10^{-8}$ cm^2 s^{-1}). The inset is the enlargement of dotted-line area.

References

1 a) N. Plumeré, O. Rudiger, A. A. Oughli, R. Williams, J. Vivekananthan, S. Poller, W. Schuhmann and W. Lubitz, *Nat. Chem.*, 2014, **6**, 822; b) A. A. Oughli, F. Conzuelo, M. Winkler, T. Happe, W. Lubitz, W. Schuhmann, O. Rüdiger and N. Plumeré, *Angew. Chem., Int. Ed.*, 2015, **54**, 12329.
2 Darren Buesen, Huaiguang Li, Nicolas Plumeré, to be submitted 2018.
3 E. Laviron, *J. Electroanal. Chem. Interfacial Electrochem.*, 1979, **100**, 263.
4 C. P. Andrieux and J. M. Savéant, *J. Phys. Chem.*, 1988, **92**, 6761.
5 V. Fourmond, S. Stapf, H. Li, D. Buesen, J. Birrell, O. Rudiger, W. Lubitz, W. Schuhmann, N. Plumeré and C. Léger, *J. Am. Chem. Soc.*, 2015, **137**, 5494.
